# Systematic Literature Review on the Incidence and Prevalence of Heart Failure in Children and Adolescents

**DOI:** 10.1007/s00246-017-1787-2

**Published:** 2017-12-20

**Authors:** Robert E. Shaddy, Aneesh Thomas George, Thomas Jaecklin, Eimear Nic Lochlainn, Lalit Thakur, Rumjhum Agrawal, Susan Solar-Yohay, Fabian Chen, Joseph W. Rossano, Thomas Severin, Michael Burch

**Affiliations:** 1 Children’s Hospital Los Angeles, 4650 Sunset Blvd. MS#126, Los Angeles, CA 90027 USA; 2 Novartis Healthcare Pvt. Ltd., Hyderabad, India; 3 Shire International GmbH, Global Clinical Development, Zählerweg 10, 6300 Zug, Switzerland; 4 Novartis Pharma AG, Basel, Switzerland; 5 Novartis Pharmaceuticals Corporation, East Hanover, NJ USA; 6 Children’s Hospital of Philadelphia, University of Pennsylvania Perelman School of Medicine, Philadelphia, PA USA; 7 Cardiorespiratory Division, Great Ormond Street Hospital for Children, London, UK

**Keywords:** Pediatric, Heart failure, Systematic, Prevalence, Incidence, Epidemiology

## Abstract

While the epidemiology of adult heart failure has been extensively researched, this systematic review addresses the less well characterized incidence and prevalence of pediatric HF. The search strategy used Cochrane methodology and identified 83 unique studies for inclusion. Studies were categorized according to whether the HF diagnosis was reported as primary (*n* = 10); associated with other cardiovascular diseases (CVDs) (*n* = 49); or associated with non-CVDs (*n* = 24). A narrative synthesis of the evidence is presented. For primary HF, the incidence ranged from 0.87/100,000 (UK and Ireland) to 7.4/100,000 (Taiwan). A prevalence of 83.3/100,000 was reported in one large population-based study from Spain. HF etiology varied across regions with lower respiratory tract infections and severe anemia predominating in lower income countries, and cardiomyopathies and congenital heart disease major causes in higher income countries. Key findings for the other categories included a prevalence of HF associated with cardiomyopathies ranging from 36.1% (Japan) to 79% (US); associated with congenital heart disease from 8% (Norway) to 82.2% (Nigeria); associated with rheumatic heart diseases from 1.5% (Turkey) to 74% (Zimbabwe); associated with renal disorders from 3.8% (India) to 24.1% (Nigeria); and associated with HIV from 1% (US) to 29.3% (Brazil). To our knowledge, this is the first systematic review of the topic and strengthens current knowledge of pediatric HF epidemiology. Although a large body of research was identified, heterogeneity in study design and diagnostic criteria limited the ability to compare regional data. Standardized definitions of pediatric HF are required to facilitate cross-regional comparisons of epidemiological data.

**Electronic supplementary material:**

The online version of this article (10.1007/s00246-017-1787-2) contains supplementary material, which is available to authorized users.

## Introduction

Heart failure (HF) is recognized as a complex clinical syndrome associated with a wide range of abnormalities in cardiac structure or function. Although definitions can vary [1–4], HF can be broadly described as “the failure of the heart to supply blood to either systemic or pulmonary circulation at an appropriate rate of flow, or to receive venous return at an appropriate filling pressure, resulting in adverse effects on the heart, the circulation, and the patient” [4].

While the epidemiology of HF has been extensively researched in the adult population [5], the incidence and prevalence of pediatric HF is not as well characterized. The most common causes of adult HF, which include ischemia, hypertension, and valvular inflammation, rarely occur in children [6]. Furthermore, existing evidence shows that the etiology of pediatric HF varies across regions and this variation affects the inter-regional incidence and prevalence of HF in children and adolescents. According to a 2009 World Health Organization (WHO) report, the main causes for HF in children are congenital malformations, cardiomyopathy and anthracycline toxicity [7]. In lower income countries, many cases of HF are caused or exacerbated by anemia which is often secondary to malaria or malnutrition [7]. Moreover, the WHO report also identifies hypocalcemia and vitamin D deficiency as risk factors for HF among children and adolescents of certain ethnic minorities in developed countries [7]. Etiologies affecting the incidence and prevalence of HF also vary according to age [8]. These factors may explain the current lack of a globally accepted definition of, and standard diagnostic criteria for, pediatric HF [6–9]. In addition, the current understanding of the epidemiology of HF in children and adolescents is poor and this topic has not been assessed in a systematic way.

We report a systematic review and narrative synthesis of the evidence on the incidence and prevalence of HF in children and adolescents (birth to < 18 years of age) over the last 20 years (1996–2016) to strengthen current knowledge on the epidemiology of pediatric HF, which can be helpful in the development of new treatments and guidelines for this patient population.

## Methods

The systematic literature review was conducted using standard methodology as published by the Cochrane Collaboration [10] and was reported in line with the Preferred Reporting Items for Systematic Reviews and Meta-Analyses (PRISMA) guidelines [11].

A full description of the multi-string search strategy is presented in Supplementary Appendix and included a disease term (heart failure/insufficiency or cardiac or myocard*); a population term (pediatric* or paediatric* or neonat* or perinat* or child* or juvenile* or bab* or infant* or toddler* or newborn or new-born or premature* or preterm* or pre-term* preschool* or pre-school* or teen* or adolescen* or minor* or pubescen*); and an outcome term (prevalen* or inciden*).

The review included observational studies. Titles, abstracts, and full-text articles were independently screened for inclusion by two reviewers and any discrepancies were reconciled by a third independent reviewer.

Data on incidence and/or prevalence of HF, and the distribution of HF in various subgroups were extracted by one reviewer, quality checked by the second reviewer, with differences reconciled by a third reviewer. Full-text studies were graded for quality according to the Downs and Black checklist (studies that scored ≤ 14 points were ranked as ‘poor’; 15–19 points as ‘fair’; and 20–25 points as ‘good’) [12]. Conference abstracts inherently lack information on many parameters listed in the checklist and, therefore, were not graded. For uniformity, we have used the term HF for all studies that report the condition as HF, chronic HF (CHF), or congestive HF and used the term acute HF (AHF) for studies that report the condition as decompensated HF or AHF, in the text. The extracted data from all the included studies are presented in Supplementary Appendix. The systematic review protocol is available in Supplementary Appendix.

## Results

### Study Selection

**Fig. 1:**
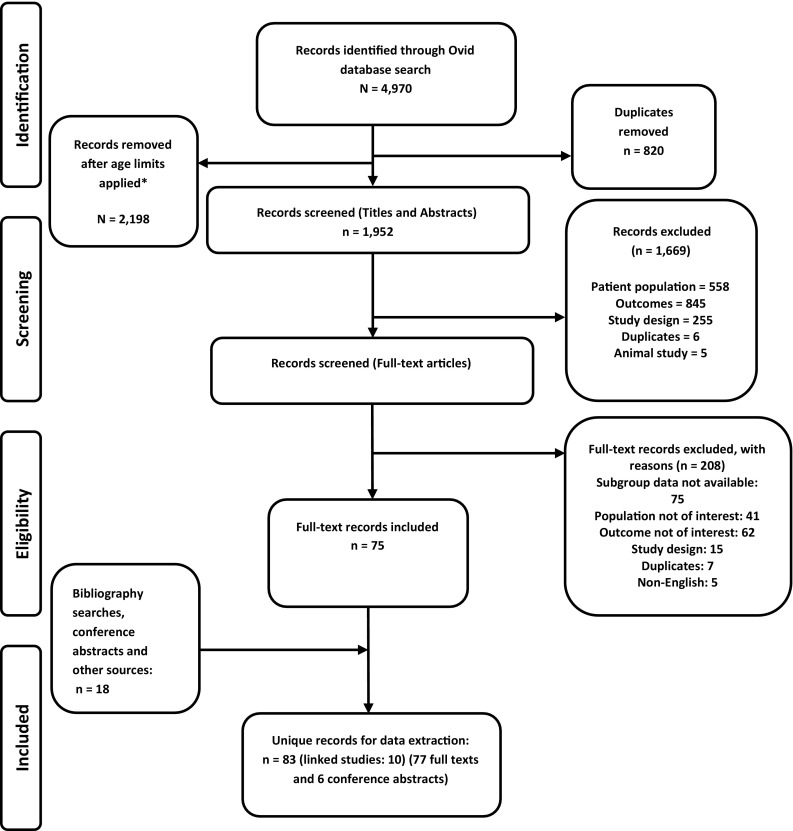
PRISMA flowchart for study selection for the systematic review. * Age-specific limits applied to EMBASE were (infant < to 1 year > or child < unspecified age > or preschool child < 1–6 years > or school child < 7–12 years > or adolescent < 13–17 years >). Age-specific limits applied to MEDLINE were limit 19 to [“all infant (birth to 23 months)” or “all child (0–18 years)” or “newborn infant (birth to 1 month)” or “infant (1–23 months)” or “preschool child (2–5 years)” or “child (6–12 years)” or “adolescent (13–18 years)”]

A final list of 1952 records was generated following the removal of duplicate records, and the application of age limits (< 18 years in EMBASE) and/or definitions for children and adolescents (EMBASE and MEDLINE). From this list, a total of 83 unique records (77 full-text publications and six conference abstracts) were selected for inclusion (see PRISMA flowchart Fig. 1). Study quality was graded as ‘poor’ for 63 and ‘fair’ for 14 of the 77 full-text studies.

To account for a lack of disease homogeneity, the included studies were grouped into the following three disease categories: studies in which (1) HF was the primary diagnosis; (2) HF was diagnosed secondary to another cardiovascular disease (CVD); (3) HF was diagnosed secondary to a non-CVD. The results are presented separately for each category.

Summary tables are presented for each category. In addition, tables summarizing all data extracted for each included study, and encompassing data on all subgroups and regional distributions, are presented in Supplementary Appendix.

### Primary HF Diagnosis

#### Incidence

Incidence was reported in 5 studies, 4 of which were multi-center studies [13–16], 2 were prospective [13, 17], and 3 retrospective [14–16]. Incidence data ranged from 0.87 per 100,000 population in a study in the United Kingdom (UK) and Ireland [13] to 7.4 per 100,000 population in a study from Taiwan (Table 1) [16].

**Table 1:** Incidence and prevalence of HF in studies on primary HF diagnosis

Study name	Study design	Country, period	Setting	Study population (age range)	Subgroups	Type of HF	Sample size	Gender		Cases (n)	(%)	Per 100,000
								Female (n)	Female (%)			
Incidence of HF as a primary diagnosis												
Andrews [13]	Prospective	UK and Ireland, 2003 (1 year)	Hospitals (17)	Hospitalized HF cases (0–16 years)	All patients	HF	11,712,100*	NR	NR	104	NR	0.87
Massin [17]	Prospective	Belgium, 1996–2006 (10 years)	Hospital (1)	Hospitalized cases (0–16 years)	All patients	HF	1196	620	51.8*	124	10.4	–
Neumann [14]	Retrospective	Germany, 2000–2006 (7 years)	Country wide hospitals	Hospitalized HF cases (0–< 15 years)	Years 2000–2006 (7 years)	HF	NR	NR	NR	NR	NR	2.0–3.0
Schmidt [15]	Retrospective	Germany, 1995 and 2009 (2 distinct years)	Country wide hospitals	Hospitalized HF cases	Year 1995	HF	1,32,38,000	NR	NR	265	NR	2
					Year 2009	HF	1,10,30,000	NR	NR	221	NR	2
Tseng [16]	Retrospective	Taiwan, 2005 (1 year)	Country wide hospitals	Hospitalized HF cases (0–14 years)	All patients	HF	190,362*	90,873	47.7	14*	NR	7.4*
					0–4 years	HF	55,262	26,319	47.6	12	NR	21.7
					5–9 years	HF	65,636	31,355	47.8	0	NR	0
					10–14 years	HF	69,464	33,199	47.8	2	NR	2.9
				Males	0–14 years	HF	99,489*	–	–	6	NR	6.0*
				Females	0–14 years	HF	90,873*	90,873	100	8*	NR	8.8*
Prevalence of HF as a primary diagnosis												
Adekanmbi [18]	Prospective	Nigeria, 2002–2003 (1 year)	Hospital (1)	Hospital admissions and ER	All patients	Congestive HF	1552	NR	NR	109	7	–
				(1 day–14 years)								
Animasahun [19]	Prospective	Nigeria, 2011–2012 (2 years)	Hospital (1)	Hospital admissions	All patients	Congestive HF	5705	NR	NR	156	2.7	–
				(1 day–12 years)								
Jiménez-García [20]	Cross-sectional	Spain, 2012–2013 (1 year)	Community (Madrid)	Influenza vaccination coverage (6 months–14 years)		HF	9,81,855	4,77,928	48.7	818	0.1	83.3*
Rodríguez-Rieiro [21]		Spain, 2009 (point prevalence)			Patients with chronic diseases	HF	1,17,940	48,806	41.4	689	0.6*	77^$^
Lagunju [22]	Prospective	Nigeria, 2000–2001 (10 months)	Hospital (1)	Hospital admissions (8 days–12 years)	All patients	Congestive HF	1713	NR	NR	100	5.8	–
Oyedeji [23]	Prospective	Nigeria, 2007 (6 months)	Hospital (1)	Patients in ER (1 month–12 years)	All patients	Congestive HF	391	NR	NR	35	9	–

*ER* emergency room, *HF* heart failure, *NR* not reported

*Calculated values from the source article

^$^Reported as prevalence of 7.7 per 10,000 inhabitants

In the UK and Ireland study undertaken in 2003, the majority of pediatric HF patients (55.8%) had HF associated with familial or idiopathic dilated cardiomyopathies, with 82% of the patients having New York Heart Association (NYHA) class III–IV severity of HF [13]. The incidence varied by regions within the UK and Ireland, with the highest incidence in Scotland and lowest in Ireland (1.27 and 0.11 per 100,000, respectively) (Supplementary Appendix, Table A1) [13].

The incidence of HF was 10.4% in 1196 patients aged 0–16 years (60% of whom were infants) primarily diagnosed with congenital or acquired heart disease and prospectively indexed at a single center in Belgium over a 10-year period (Table 1) [17]. Congenital heart disease was the HF etiology in 52% of patients, cardiomyopathies in 19.4%, and acquired heart disease in 18.5% (Supplementary Appendix, Table A1).

Two German studies reported on the nationwide incidence of HF hospitalizations [14, 15]. According to the first study, the incidence of hospitalized HF ranged from 2 to 3 per 100,000 population among children and adolescents (aged 0 to < 15 years; period covered from 2000 to 2006) [14]. A similar incidence of hospitalized HF of 2 per 100,000 population was reported in the second German study over two distinct 1-year periods in 1995 and 2009 [15].

A 2005 study from Taiwan reported an incidence of hospitalized HF of 7.4 per 100,000 pediatric patients aged 0–14 years. The incidence was slightly higher among girls versus boys (8.8 vs. 6 per 100,000, respectively) and was highest in the 0–4 year age group (21.7 per 100,000 population) [16] (Table 1).

#### Prevalence

Prevalence data were obtained from 5 unique studies comprising one large population-based study from Spain [20, 21] and 4 smaller studies from different university hospitals in Nigeria [18, 19, 22, 23].

In a 2009 study conducted to determine the extent of influenza vaccine coverage in chronically ill patients in Madrid, the prevalence of HF in 117,940 pediatric patients was 0.6% (77 per 100,000 inhabitants) (Table 1) [21]. In a subsequent 2012–2013 publication using the same computerized immunization registry, but not restricted to chronically ill patients, a HF prevalence of 0.1% (83.3 per 100,000) was reported among 981,855 children aged 6 months–14 years [20].

The four hospital-based studies from Nigeria reported a pediatric HF prevalence ranging from 2.7 to 9% in studies of children presenting at emergency rooms or admitted to pediatric hospital wards (Table 1) [18, 19, 22, 23]. The highest prevalence was observed in the youngest age group (1 month–5 years) (Supplementary Appendix, Table A2). The most common HF etiologies in these studies were anemia and respiratory tract infections (Supplementary Appendix, Table A3) [18, 19, 22, 23].

### Secondary HF Diagnosis in CVDs

HF as a diagnosis secondary to other CVDs was reported in 49 of 83 identified studies. Five studies reported HF incidence alone, 42 studies reported HF prevalence only, and 2 studies had both incidence and prevalence data (Table 2).

**Table 2:** Incidence of HF secondary to other CVDs

Study name	Study design	Country, period	Setting	Study population (age range)	Subgroups	Type of HF	Sample size	Gender		HF incidence	
								Female (n)	Female (%)	Cases (n)	Incidence (%)
Congenital heart defects/disease											
Hong [24]	Retrospective	South Korea, 2000–2010 (11 years)	Hospital (1)	TGA (1–108 days)	All patients	HF	28	11	39.3	5	17.9
Najm [25]	Retrospective	Canada, 1975–1985 (21 years)	Hospital (1)	Scimitar syndrome (1–335 days)	–	HF	19	14	73.7*	11	57.9*
Tomlinson [26]^#^	Retrospective	Jamaica, 1995–2004 (10 years)	Hospital (1)	Trisomy 21 with congenital heart disease and cardiac lesions (0–12 years)	–	Congestive HF	46	NR	NR	11*	23.9*
Vascular malformations											
Rialon [27]	Retrospective	US, 1995–2012 (18 years)	Hospital (1)	Hepatic hemangiomas (0 to < 1 year)	All patients	Congestive HF	72	NR	NR	16*	22.2*
					Patients who underwent initial screening for hemangiomas	Congestive HF	43	NR	NR	2	5
					Unscreened patients	Congestive HF	29	NR	NR	14	48
Post-OHT											
LaPage [28]^$^	Retrospective	US, 1991–2006 (16 years)	Hospital (1)	Tachyarrhythmia (0–17 years)		Acute congestive HF	19	NR	NR	2*	10.5*
Murtuza [29]	Retrospective	UK, 2000–2011 (8 years)	Hospital (1)	DCM and RCM (0.1–17.1 years)	All patients	VHF (right)	159*	83*	52.2*	30*	18.9
					Patients with DCM	VHF (right)	136	74*	54.4*	20	14.7
					Patients with RCM	VHF (right)	23	9*	39.1*	10	43.5
IE											
Marom [30]^$^	Retrospective	Israel, 1992–2004 (12.5 years)	Hospital (1)	IE (0 to < 18 years)	Children with no predisposing factors for IE	HF	9	NR	NR	7	77.8

*DCM* dilated cardiomyopathy, *HF* heart failure, *IE* infective endocarditis, *NR* not reported, *OHT* orthotopic heart transplantation, *RCM* restrictive cardiomyopathy, *VHF* ventricular heart failure

*Calculated from source article

^#^In Tomlinson et al., 30 of the 76 children had congestive HF at presentation and this is captured in “Prevalence,” and in 11 of the remaining 46 children congestive HF developed during the study. A total of 41 patients (30 + 11) had congestive HF in this study

^$^In Marom et al., 9 of a total of 51 patients with IE had no predisposing cardiac anomalies (HF cases are new). Of these, 7 cases had HF and have contributed to incidence data, whereas 42 patients had predisposing cardiac anomalies (unclear if HF cases are new) and the data are captured in “Prevalence”

#### Incidence

##### Congenital Heart Disease

Three retrospective studies reported the incidence of HF in pediatric patients diagnosed with congenital heart disease [24–26]. A Canadian study reported a HF incidence of 57.9% among 19 infants with Scimitar Syndrome (Table 2) [25]. In a Jamaican study, HF developed in 23.9% of 46 patients with trisomy 21 and congenital heart disease and/or cardiac lesions [26]. A study from South Korea reported that, overall, HF developed in 17.9% of 28 patients presenting with transposition of the great arteries (TGA), and the rate was 41.7% in patients who also had ventricular septal defects (VSDs) (Table 2) [24].

##### Vascular Malformations

In a retrospective study from the US covering 1995–2012, HF developed in more than 20% of the 72 infants with multiple cutaneous and hepatic hemangiomas (Table 2) [27]. The incidence of HF was lower among patients identified through screening for hemangiomas (5% of 43 vs. 48% of 29 not screened) [27].

##### Post-orthotopic Heart Transplantation

Two retrospective studies (one UK- and one US-based) reported the incidence of HF in post-orthotopic heart transplantation (OHT) pediatric recipients [28, 29]. In the UK-based study, 18.9% of 159 patients developed right ventricular heart failure (VHF) during the perioperative period. Complex congenital heart disease, restrictive cardiomyopathy (RCM), and dilated cardiomyopathy (DCM) were the main reasons for OHT in these populations. The incidence of HF was 43.5% in 23 RCM patients and 14.7% in 136 DCM patients (Table 2) [29]. The US study reported that acute congestive HF developed in 10.5% of 19 patients (0–17 years) who presented with tachyarrhythmia beyond the first 2 weeks post-OHT (Table 2) [28].

##### Infective Endocarditis

HF is one of the many complications of infective endocarditis (IE). A retrospective study from Israel reported incident cases of HF occurring in 77.8% of 9 children with IE, without any predisposing factors [30].

#### Prevalence

Due to the large number of studies included for the prevalence of HF secondary to other CVDs, only those that ranked ‘fair’ or ‘good’ on the Downs and Black checklist and/or had a sample size > 50 and/or report acute HF are summarized in the text below and listed in Table 3. However, a consolidated table of all included studies is presented in Supplementary Appendix, Table B2.

**Table 3:** Prevalence of HF in CVD studies

Study name	Study design	Country, period	Setting	Study population (age range)	Subgroups	Type of HF	Sample size	Gender		HF prevalence and distribution in study subgroups		
								Female (n)	Female (%)	Cases (n)	Prevalence (%)	Distribution of prevalent cases of HF in study subgroups (%)
Congenital heart defects/disease												
Azhari [31]	Retrospective	Saudi Arabia, 1990–2003 (14 years and 1 month)	Hospital (1)	ASD (1 day–11 years)	All patients	Congestive HF	121	74	61.2	14	11.6	–
					Small defects	Congestive HF	22	9*	41	0	0	–
					Medium defects	Congestive HF	27	NR	NR	1	3.7*	–
					Large defects	Congestive HF	72	NR	NR	13	18.1*	–
					Pulmonary arterial hypertension^@^	Congestive HF	8	2	25	8	100*	–
Meberg [32]	Longitudinal (prospective and retrospective)	Norway, 1982–1996 (15 years)	Hospitals (NR)	Congenital heart disease (2 weeks–11 years)	Detected subsequent to discharge from hospital after birth	Decompensation	84	NR	NR	7	8.0	–
Miyake [33]	Prospective	Japan, 1986–1996 (11 years)	Hospital (1)	VSD (1–88 days)	All patients	Congestive HF	225	109	48.4*	104	46.0	–
					Subpulmonary VSD	Congestive HF	104	NR	NR	18*	–	17.3*
					Perimembranous VSD	Congestive HF	104	NR	NR	85	–	81.7*
					Muscular	Congestive HF	104	NR	NR	1	–	1*
					Spontaneous closure	Congestive HF	104	NR	NR	20	–	19.2*
					Small open	Congestive HF	104	NR	NR	31	–	29.8*
					Surgical closure	Congestive HF	104	NR	NR	53	–	51*
Najm [34]	Retrospective	Canada, 1982–1996 (14 years and 5 months)	Hospital (1)	ASD (1 month–16.4 years)	–	Congestive HF	180	97	53.9	35	20	–
Okoromah [35]	Case–control	Nigeria, 2006–2008 (2 years)	Cases; hospital (1)	Cases: malnutrition and congenital heart disease (3–192 months)	All cases	Congestive HF	73	NR	NR	60	82.2	–
			Controls; community (primary school)	Controls: malnutrition with no congenital heart disease (3–192 months)	All controls	Congestive HF	76	NR	NR	0	0	–
Sadoh [36]	Prospective	Nigeria, 2006–2009 (2 years and 5 months)	Hospital (1)	VSD (2–24 months)	All patients	Congestive HF	61	35	57.4	15	24.6	–
					Spontaneous closure	Congestive HF	15	NR	NR	3	–	20
Sadoh [37]	Prospective	Nigeria, 2011–2012 (1 year)	Hospital (1)	Pneumonia with and without congenital heart disease (1–48 months)	All patients	Congestive HF	121	60	49.6	49	40.5	–
					Pneumonia and congenital heart disease	Congestive HF	14			9	64.3	–
					Pneumonia without congenital heart disease	Congestive HF	107			40	37.4	–
Shah [38]	Retrospective	Nepal, 2006 (1 year)	Hospital (1)	Congenital heart disease (0 to < 15 years)	–	Congestive HF	84	33	39.3	46	54.8	–
Tomlinson [26]	Retrospective	Jamaica, 1995–2004 (10 years)	Hospital (1)	Trisomy 21 with congenital heart disease (0–12 years)	–	Congestive HF	76	46	60	30	39.5*	–
Vaidyanathan [39]	Prospective	India, 2005–2006 (1 year)	Hospital (1)	Malnutrition with congenital heart disease (0 to < 5 years)	–	Congestive HF	476	243*	51.5*	194	40.8	–
Cardiomyopathies												
Alvarez [40], Colan [41], Everitt [42], Towbin [43], Webber [44], Wilkinson [45] (PCMR studies)	Longitudinal (prospective and retrospective cohorts)	US, Canada, 1990 (ongoing)	Hospitals (98 centers for the prospective cohort and 39 centers for the retrospective cohort)	Cardiomyopathies (0 to < 18 years)	All patients	Congestive HF	3549^±^	NR	NR	NR	NR	–
					All HCM patients	Congestive HF	849	NR	NR	115*	13.5*	–
					Inborn errors of metabolism	Congestive HF	74	NR	NR	30*	40.3	–
					Malformation syndromes	Congestive HF	77	NR	NR	18*	23.4	–
					Neuromuscular disorders	Congestive HF	64	NR	NR	4*	6.4	–
					Infantile/ idiopathic	Congestive HF	634	NR	NR	63*	9.9	–
		US, Canada, 1990–2007 (18 years)		DCM (0 to < 18 years)	All DCM patients	Congestive HF	1682	777*	46.2*	1,205*	71.6*	–
					Idiopathic DCM	Congestive HF	1192	599*	50.2	894	75	–
					Neuromuscular disease	Congestive HF	139	5*	3.6*	40	28.8	–
					Familial isolated DCM	Congestive HF	79	35*	44.3*	44	55.7	–
					Myocarditis	Congestive HF	272	138*	51*	227	83.4	–
		US, Canada, 1990–2008 (19 years)		RCM (0 to < 18 years)	All RCM patients	Congestive HF	152	79*	52*	56*	37	–
					Pure RCM	Congestive HF	101	51*	51*	42*	42	–
					RCM/HCM	Congestive HF	51	27*	53*	13*	26	–
Nugent [46]	Retrospective	Australia, 1987–1996 (10 years)	Hospitals (21)	Cardiomyopathies (0 to < 10 years)	All patients	Congestive HF	314	148*	47.1*	206*	65.6*	–
					DCM	Congestive HF	184	103	56	165	89.7	–
					HCM	Congestive HF	80	25	31.2	6	7.5	–
					RCM	Congestive HF	8	4	50	4	50	–
					Unclassified cardiomyopathy	Congestive HF	42	16	38.1	31	73.8	–
Saji [47]	Retrospective	Japan, 1997–2002 (6 years)	Hospitals (65)	Myocarditis (1 month–17 years)	All patients	HF	169	NR	NR	61	36.1	–
					Fulminant myocarditis	HF	64	NR	NR	34	53.1	–
					Acute myocarditis	HF	89	NR	NR	27	30.3	–
					Chronic myocarditis	HF	8	NR	NR	NR	NR	
					Myocarditis of unknown type	HF	8	NR	NR	NR	NR	
Soongwang [48]	Retrospective	Thailand, 1996–2000 (5 years)	Hospitals (5)	Myocardial diseases (0.1–14.5 years)	All Patients	Congestive HF	209	117*	56.0	151*	72.0	–
					DCM	Congestive HF	94	51	54.3	79	84.1	–
					Acute myocarditis	Congestive HF	57	38	66.7	45	78.9	–
					HCM	Congestive HF	38*	18	47.4	17	44.7	–
					Hypertrophic obstructive cardiomyopathy	Congestive HF	17*	8	47.1	8	47.1	–
					RCM	Congestive HF	3	2	66.7	2	66.6	–
Tsirka [49]	Retrospective	US, 1990–1999 (10 years)	Hospitals (2)	DCM (0–17.8 years)	–	Congestive HF	91	33*	36.3*	72	79	–
Rheumatic fever/rheumatic heart disease												
Bitar [50]	Retrospective	Lebanon, 1980–1995 (16 years)	Hospital (1)	RF (3–17 years)	–	Acute congestive HF	91	38*	42*	40*	44	–
da Silva [51]	Retrospective	Brazil, 1989–1994 (6 years)	Hospitals (7)	RF (3–17 years)	–	HF	786	382	48.7	119	15.1*	–
Gapu [52]	Cross-sectional	Zimbabwe, 2012–2013 (11 months)	Hospitals (2)	Acute RF and/or RHD (1–12 years)	All patients	Any HF	50	32	64.0	37*	74*	–
					Outpatients	Chronic HF	19	NR	NR	15	78.9	–
					Hospitalized children with acute RF and/or RHD	Congestive HF	31	NR	NR	22	71.0*	–
					Hospitalized with RHD only	AHF	22	NR	NR	20	–	90.9
					Hospitalized with acute RF only	AHF	9	NR	NR	2	–	22.2
Karlassan [53]	Retrospective	Turkey, 1993–1998 (5 years)	Hospital (1)	Acute RF (5–17 years)	–	Congestive HF	274	147	53.6	4	1.5*	–
Örün [54]	Retrospective	Turkey, 1980–2009 (30 years)	Hospital (1)	Acute RF (2–15 years)	–	HF	1115	510	45.8	100	9.0*	–
Qurashi [55]	Longitudinal (retrospective and prospective)	Saudi Arabia, 1994–2003 (10 years)	Hospital (1)	Acute RF (4–12 years)	–	HF	83	NR	NR	14	16.9*	–
Rayamajhi [56]	Prospective	Nepal, 2003–2005 (2 years)	Hospital (1)	Acute RF (5–14 years)	–	HF	51	NR	NR	14	28	–
IE												
Lertsapcharoen [57]	Retrospective	Thailand, 1987–2004 (18 years)	Hospital (1)	IE (2 months–15 years)	–	Congestive HF	57	28	49.1*	15	26	–
Marom [30]	Retrospective	Israel, 1992–2004 (12.5 years)	Hospital (1)	IE (0 to < 18 years)	Children with predisposing factors for IE^€^	HF	42	NR	NR	10	23.8	–
Sadiq [58]	Prospective	Pakistan, 1997–2000 (4 years)	Hospital (1)	IE (4 months–16 years)	All patients	HF	45	15	33.3*	18	40	–
					Rheumatic heart disease	HF	24			10	42	–
					Congenital heart disease	HF	20			8	40	
					Myocarditis	HF	1	–	–	0	0	
Rhythm and conduction disturbances												
Massin [59]	Retrospective	Belgium, 1995–2006 (11 years)	Hospitals (3)	Tachyarrhythmia (0 to < 16 years)	All patients	HF	250	92*	36.8*	49	19.6*	
					Infants	HF	109			33	30.3*	
Others												
Borzouee [60]	Retrospective	Iran, 2001–2003 (2 years)	Hospital (1)	Cardiac problems (1 day–16 years)		HF	1817	NR	NR	25	1.4	

*ASD* atrial septal defect, *CVD* cardiovascular disease, *DCM* dilated cardiomyopathy, *IE* infective endocarditis, *HCM* hypertrophic cardiomyopathy, *HF* heart failure, *NR* not reported, *RCM* restrictive cardiomyopathy, *RF* rheumatic fever, *RHD* rheumatic heart disease, *VSD* ventricular septal defect

*Calculated data from source article

^@^In Azhari et al. [31], the patients with pulmonary arterial hypertension is inclusive pf patients with small, medium, or large defects and so is not a stand-alone group

^±^3549 is the most recent number of total patients with different cardiomyopathies (HCM, DCM, RCM) from PCMR registry studies. However, the total of HCM, DCM, and RCM does not add up to this number (Wilkinson et al. [45]). The data for HF in HCM, DCM, and RCM are taken from different PCMR publications

^€^Data on 9 children without predisposing factors in Marom et al. [30] are present in “Incidence,” so the total does not add up to 50

##### Congenital Heart Disease

The prevalence of HF in various congenital heart diseases was reported and summarized from 17 studies, and ranged from 8% of 84 patients in a study from Norway [32] to 82.2% of 73 patients from a study in Nigeria [35] (Supplementary Appendix, Table B2).

Few studies in this disease category focused on specific congenital defects, such as atrial septal defects (ASDs) or VSDs. A prospective study from India reported a prevalence of HF of 40.8% in 476 malnourished children with congenital heart disease aged < 5 years [39], demonstrating the importance of the association between malnutrition and congenital heart disease and consequent sequelae such as HF. Similarly, a Nigerian case–control study reported a prevalence of HF of 82.2% among 73 children with congenital heart disease (90.4% of these 73 children were malnourished) compared with none among 76 children without congenital heart disease (21.1% of these 76 children were malnourished) (Table 3) [35]. Another prospective study, from Nigeria, reported a 64.3% prevalence of HF among 14 children with congenital heart disease and pneumonia compared with 37.4% among 107 children without congenital heart disease, but with pneumonia (Table 3) [37].

In a retrospective, hospital-based study from Jamaica, a HF prevalence of 39.5% was found in 76 patients with trisomy 21 and congenital heart disease [26]. A Nepalese study reported a HF prevalence of 54.8% of 84 pediatric patients aged < 15 years with congenital heart disease (Table 3) [38].

A Norwegian study reported acute heart failure (AHF) as the presenting symptom in 8% of 84 pediatric patients aged 2 weeks–11 years with congenital heart disease (Table 3) [32]. Four of these patients had VSDs, one had an atrioventricular septal defect, and another had coarctation of the aorta. There was one case of endocardial fibroelastosis (Supplementary Appendix Table B2) [32].

Two prospective studies that reported on the prevalence of comorbidities, including HF, in patients with VSDs are summarized in Table 3. In a Japanese prospective study, the prevalence of HF was 46% among 225 Japanese infants < 3 months of age diagnosed with VSDs over a period of 11 years (1986–1996) [33]. HF was most prevalent in patients with perimembranous VSDs and least prevalent among patients with a defect in the muscular septum (81.7 and 1%, respectively) (Table 3). Spontaneous closure of the VSDs occurred in 19 versus 72% of the patients with and without HF, respectively, and surgical closure was required 51 versus 5% of these respective patients [33]. HF was the presenting symptom in 24.6% of the 61 Nigerian children with VSD aged 2–24 months (Table 3), of whom only 20% had spontaneous closure of the VSDs [36].

Two retrospective studies focused on pediatric patients with ASDs are summarized in Table 3. In a hospital-based study from Canada, HF was the presenting symptom in 20% of the 180 ASD patients aged 1 month–16.4 years [34] (Table 3). Another hospital-based study from Saudi Arabia reported that HF was prevalent in 11.6% of 121 ASD patients aged 1 day–11 years [31]. In the Saudi Arabian study, HF prevalence was 18.1% among patients with large defects (≥ 8 mm), 3.7% with medium defects (5–8 mm), and 0% in patients with small defects (3–5 mm) (Table 3) [31].

##### Cardiomyopathies/Myocarditis

Seven unique studies reported the prevalence of HF in myocardial diseases (cardiomyopathies and myocarditis; Supplementary Appendix, Table B2) and five are summarized below. As shown in Table 3, studies from the Pediatric Cardiomyopathy Registry (PCMR) had the largest population base regarding prevalence of pediatric HF in cardiomyopathies and contains data from multiple centers in the US and Canada. In this registry, the prevalence of HF was 71.6% among 1682 DCM patients, 37% among 152 RCM patients, and 13.5% among 849 hypertrophic cardiomyopathy (HCM) patients [40, 44, 45]. Idiopathic DCM was the most common cause of DCM, and 75% of these patients presented with HF. For HCM, the highest proportion of HF was among those with inborn errors of metabolism (40.3%). The most common etiology for HCM was idiopathic (unknown) (Table 3) [40, 45].

An overall prevalence of HF of 65.6% was reported in an Australian 21-center retrospective study of children < 10 years with different cardiomyopathies [46]. In this study, a prevalence of 89.7% was reported for 184 DCM patients, a prevalence of 50% among 8 RCM patients, and 7.5% among 80 HCM patients (Table 3) [46]. Similarly, a high prevalence of HF (79%) was also observed in 91 DCM patients, in a US-based retrospective study [49]. In another study from 5 hospitals in Thailand that included cardiomyopathy patients aged 0.1–14.5 years, HF was reported in 84.1% of 94 patients with DCM, 66.6% of 3 RCM patients, 47.1% of 17 patients with hypertrophic obstructive cardiomyopathy, and 44.7% of 38 HCM patients [48]. Additionally, HF was present in almost 80% of 57 patients with acute myocarditis [48], which contrasts with a smaller percentage reported in a Japanese study [47]. The Japanese study reported that 53.1% of the 64 patients with fulminant myocarditis had HF at admission, whereas HF was present at admission in only 30.3% of 89 patients with acute myocarditis (Table 3). In this Japanese study, the authors stated that “fulminant myocarditis represents approximately 20–30% of myocarditis cases, and can be clinically differentiated from acute myocarditis by the presence of severe hemodynamic deterioration, cardiogenic shock, severe ventricular dysfunction, and/or refractory life-threatening arrhythmias requiring inotropic support or mechanical cardiopulmonary assist devices” [47]. It is thus unclear why HF was “present” in only 53.1% of patients with fulminant myocarditis [47]. Myocarditis is often associated with viral infection and in this Japanese study, 25% (22 of 89) and 19% (12 of 64) of the total number of acute and fulminant cases were associated with viral pathogens, respectively. Coxsackie A/B and influenza were the most commonly reported infections.

##### Rheumatic Fever/Rheumatic Heart Disease

Ten studies reported the prevalence of HF in rheumatic fever (RF) and rheumatic heart disease (RHD) ranging from 1.5% in Turkey to 74% in Zimbabwe (Supplementary Appendix, Table B2) [50–56, 61–63].

The retrospective Turkish study had the largest sample size of 1115 acute RF and comprised patients admitted to a single hospital, aged 2–15 years. HF was detected in 9% of the included patients (and in 13.8% of those diagnosed with carditis), over a 30 year period (Table 3) [54]. Another retrospective study from Turkey showed that HF was the presenting symptom in only 1.5% of 274 patients with acute RF (Table 3) [53].

Among all the included studies, the cross-sectional study from Zimbabwe reported the highest proportion of patients with any HF (74% of 50 included patients) among patients with acute RF or RHD. In this study, AHF was present in 71% of the 31 hospitalized patients, and HF was detected in 78.9% of the 19 children seen in outpatient clinics (Table 3) [52]. AHF was reported in 44% of the 91 RF patients at initial presentation, in a retrospective study from Lebanon [50].

##### Infective Endocarditis

HF is one of the many complications of IE. Three studies reported the incidence of HF in the pediatric population with IE, ranging from 23.8% in Israel to 40% in Pakistan (Table 3) [30, 57, 58]. The retrospective study from Israel reported HF in 23.8% of 42 IE patients who had at least one predisposing factor such as the presence of congenital or acquired heart disease, intravenous therapy within 4 weeks before the onset of endocarditis, and previous invasive procedures (Table 3) [57].

##### Other Studies

Details of a Belgian study on the prevalence of HF patients admitted for arrhythmias and an Iranian study on the prevalence of HF patients with cardiac problems are also listed in Table 3 [59, 60].

#### Secondary HF Diagnosis in Non-CVD

Of the 83 identified studies, 24 studies reported HF as secondary diagnosis in non-CVDs.

##### Incidence of HF Associated with Anthracycline Treatment, HIV/AIDS, and Pneumonia

Three retrospective studies reported an incidence of HF between 1 and 5%, following anthracycline treatment of various childhood cancers (Table 4) [64–66]. In a cohort of 808 children from the Netherlands (aged 0‒16 years), 94% of 17 cases occurred during or within the first year of anthracycline therapy [66]. In a US study, HF developed in 1% of 97 doxorubicin-treated patients aged 7 months–17 years. The one patient who developed HF received a cumulative dose of 450 mg/m^2^ doxorubicin [64]. The highest rate of 5% was reported in a Japanese study, in which 6 of patients on anthracycline developed HF. In the Japanese study, the mean total anthracycline dose received by these patients was 383 mg/m^2^ (range: 180–520) [65].

**Table 4:** Incidence and prevalence of HF in non-CVD studies

Incidence of HF secondary to non-CVDs												
Study name	Study design	Country, period	Setting	Study population (age range)	Subgroups	Type of HF	Sample size	Gender		HF incidence		
								Female (n)	Female (%)	Cases (n)	Incidence (%)	
Hematology/oncology												
Berrak [64]	Retrospective	US, 1988–1998 (10 years)	Hospital (1)	Doxorubicin for childhood cancer (7 months–17 years)	–	Congestive HF	97	38	39.2*	1	1.0*	
Godoy [65]	Retrospective	Japan, 1985–1994 (10 years)	Hospital (1)	Anthracyclines for childhood cancer (5 months–17 years)	–	Congestive HF	120	51	42.5*	6	5.0*	
van Dalen [66]	Retrospective	Netherlands, 1976–2001 (26 years)	Hospital (1)	Anthracyclines for childhood cancer (< 2 to > 16 years)	Age < 2 to 16 years	Congestive HF	808*	NR	NR	17*	2.1*	
HIV/AIDS												
Starc [67]	Prospective	US, 1990–1997 (6 years)	Hospitals (10)	Children of HIV-infected mothers (0–14 years)	Infected children with echocardiographic evaluation available (5 years of follow-up)	Congestive HF	199^#^	NR	NR	14	14 (cumulative incidence)	
Fisher [68]											7.0 (incidence)	
Lipshultz [69]				Infants of HIV-infected mothers (0 to < 28 days)	Infected infants (5 years of follow-up)	Congestive HF	93	NR	NR	4	5.1(cumulative incidence)	
Starc [70]											4.3 (incidence)	
					Uninfected infants(5 years of follow-up)	Congestive HF	463	NR	NR	1	0.2(cumulative incidence)	
											0.2 (incidence)	
Pneumonia												
llten [71]	Prospective	Turkey, NR	Hospital (1)	Acute pneumonia (2–24 months)		Congestive HF	50	14	28	7	14	

Prevalence of HF secondary to non-CVDs												
Study name	Study design	Country, period	Setting	Study population (age range)	Subgroups	Type of HF	Sample size	Gender		HF prevalence and distribution in study subgroups		
								Female (n)	Female (%)	Cases (n)	Prevalence (%)	Distribution of prevalent cases of HF in study subgroups (%)
Renal disorders												
Duzova [72]	Prospective	Turkey, 2006–2007 (1 year)	Hospitals (17)	AKI (1–18 years)	Newborn (< 1 month)	HF	154	NR	NR	15	9.7	–
Gunasekaran [73]	Prospective	India, 2013–2014 (1 year and 6 months)	Hospital (1)	ANS (1–13 years)	PIGN	Congestive HF	72	32*	44.4*	8	11.1	–
				PIGN	PSGN	Congestive HF	65	30*	46.1*	8	12.3	–
Krishnamurthy [74]	Prospective	India, 2010–2011 (10 months)	Hospital (1)	AKI (1–144 months)		Congestive HF	54	25	46.3	2	3.8	–
Sarkissian [75]	Prospective	Armenia, 1992–1996 (5 years)	Hospital (1)	Acute PIGN (1 to < 16 years)		Congestive HF	474	166*	35*	45	10	–
Vachvanichsanong [76]	Retrospective	Thailand, 1984–2007 (26 years)	Hospital (1)	AKI (0–30 days)	All patients	Congestive HF	139	51	36.7*	17*	12.2*	–
Vachvanichsanong [77]	Retrospective	Thailand, 1982–2004 (22 years and 10 months)	Hospital (1)	Acute renal failure (1 month–16.7 years)		HF	311	NR	NR	26	8.4	–
Wong [78]	Prospective	New Zealand, 2007–2009 (2 years)	Hospitals (country wide)	Acute PSGN (definite/probable) (1.4–14.7 years)		Congestive HF	176	62	35.2*	8	4.5*	–
HIV/AIDS												
Cunha [79]	Retrospective	Brazil, 1990–2002 (13 years)	Hospital (1)	AIDS (0 to < 13 years)		Congestive HF	93	47	50.5	12	12.9*	–
Diogenes [80]	Prospective	Brazil, 1996–2004 (8 years)	Hospital (NR)	HIV-1 (13 days–13 years)	HIV infected	Congestive HF	41	NR	NR	12	29.3*	–
					HIV seroconverted	Congestive HF	43	NR	NR	0	0	–
					Dilated cardiomyopathy (as etiology for congestive HF in HIV)	Congestive HF	12			5		41.7*
Okoromah [81]	Case–control	Nigeria, 2004–2007 (3 years)	Hospital (1)	HIV positive (18–144 months)		Congestive HF	83	NR	NR	10	12	–
			Community and hospital	HIV negative (18–144 months)		Congestive HF	83	NR	NR	0	0	–
Starc [67]	Prospective	US, 1990 to Jan 1997 (6 years)	Hospitals (10)	Children of HIV-infected mothers (0–14 years)		Congestive HF	201	NR	NR	2	1	–
Fisher [68]												
Lipshultz [69]												
Starc [70]												
Hematology/oncology												
Karimi [82]	Cross-sectional	Iran, 2007–2010 (3 years)	Hospital (1)	BTM (1–15 years)	All patients	Congestive HF	328	NR	NR	47*	14.3*	–
Other conditions												
Ahmed [83]	Retrospective	Scotland, 2002–2008 (6 years)	Hospital (1)	Vitamin D deficiency (2 weeks–14 years)		HF	160	77	48.1	1	0.6	–
Camilla [84]	Cross-sectional (Pt prevalence)	Italy	Community	Organ failure (0 to < 18 years)	All inhabitants	CHF	6,47,727	NR	NR	21	0.0032*	–
					DCM (as etiology)		21	NR	NR	13		62*
Lagunju [85]	Retrospective	Nigeria, 2000–2004 (5 years)	Hospital (1)	Measles (4 months–10 years)		HF	666	319	47.9	2	0.3	–

*ANS* acute nephrotic syndrome, *AIDS* acquired immunodeficiency syndrome, *AKI* acute kidney injury, *APGN* acute post-infectious glomerulonephritis, *APSGN* acute post-streptococcal glomerulonephritis, *BTM* β-thalassemia major, *CHF* chronic heart disease, *CVD* cardiovascular disease, *DCM* dilated cardiomyopathy, *HF* heart failure, *HIV* human immunodeficiency virus, *NR* not reported, *PIGN* post-infectious glomerulonephritis, *PSGN* post-streptococcal glomerulonephritis

*Calculated data from source article

^#^In Starc et al., 2 of the 201 children had congestive HF at presentation and this is captured in “Prevalence,” and in the remaining 199 children congestive HF developed during the study

Multiple publications from the US-based P^2^C^2^ HIV study reported the incidence of HF in children of HIV-infected mothers (Table 4) [67–70]. The study categorized children into two groups: group 1 included 199 vertically infected children aged 0.1–14 years with echocardiographic evaluations and group 2 included newborns (93 HIV-infected and 463 uninfected). In group 1, a 5-year cumulative HF incidence of 14% was reported during the 5-year follow-up. In group 2, a 5-year cumulative HF incidence of 5.1 versus 0.2% was reported among the infected and uninfected infants, respectively (Table 4) [67].

In a further prospective Turkish study, 14% of 50 children aged 2–24 months with pneumonia developed HF (Table 4) [71].

##### Prevalence of HF Associated with Renal Disorders, HIV/AIDS, and Other Conditions

Nine studies reported the HF prevalence in pediatric patients with renal disorders (Supplementary Appendix, Table C2) [72–78, 86, 87]. The prevalence of HF ranged from 3.8% among patients with acute kidney injury (AKI) [74] to 24.1% among those with a primary diagnosis of acute glomerulonephritis (AGN) [87].

HF was diagnosed in 4.5‒11.1% of pediatric patients with acute post-infectious glomerulonephritis (PIGN) [73, 75, 78] and was the most common extra-renal diagnosis in a prospective study from Armenia (10% of 474 pediatric patients (Table 4)) [75]. A large prospective multi-center study from Turkey reported HF as prevalent in 9.7% of 154 children with AKI aged < 1 month old [72], while a prospective study from a hospital in India reported that 3.8% of 54 AKI patients had underlying HF [74]. Two studies from Thailand reported HF as a cause of AKI and acute renal failure in pediatric patients. The first study reported HF as present 12.2% of 139 AKI patients aged ≤ 30 days [76], whereas the second study reported HF in 8.4% of 311 acute renal failure patients aged 1 month–16.7 years (Table 4) [77].

The prevalence of HIV/AIDS patients presenting with HF ranged from 1% in the US [67] to 29.3% in Brazil (Supplementary Appendix, Table C2, Table 4) [80]. Of note, a Brazilian study reported that more than 25% of 41 HIV-infected pediatric patients had HF versus none in 43 HIV-negative patients and that DCM was the main etiology in 41.7% of these HF patients [80].

One study from Iran reported that HF accounted for 14.3% of 328 hospital admissions in β-thalassemia major patients (Table 4) [82]. In other studies, a HF prevalence of 0.3% of 666 patients was reported from a study of the complications of measles [85], 0.6% of 160 (one patient) with vitamin D deficiencies [83], and 5.3% of 38 with foreign body aspiration [88]. Of note, one population-based cross-sectional study carried out to determine the epidemiology of childhood chronic organ failure reported a prevalence of chronic HF of 0.0032%, for 647,727 inhabitants aged < 18 years. Furthermore, DCM was the main cause of HF, being the etiology in 62% of these patients (Table 4) [84].

## Discussion

This systematic review and narrative synthesis collates the existing evidence on the incidence and prevalence of HF in the pediatric population (< 18 years) and strengthens the current knowledge on the epidemiology of pediatric HF.

In studies reporting HF as a primary diagnosis, there appears to be a relatively higher incidence of HF in Taiwan (7.4 per 100,000 population) [16] compared with the European (0.87–3 per 100,000 population) pediatric population [13–15]. Possible reasons for the variation in the reported incidence rates include different definitions of HF used across studies, statistical methods (crude incidence [16] versus adjusted incidence [14, 15] rates reported), definitions of the study populations (e.g., defined population such as children with ‘heart muscle disease’ (cardiomyopathy/myocarditis, etc.) [13] versus overall HF diagnosis rates [14–16]). Furthermore, as the Asian data were from one single Taiwanese study, the results may not be generalizable to other regions of the Asian continent.

Variation within the same geographic regions was also apparent. The slight difference in incidence reported from Germany [14, 15] and the UK and Ireland study [13] may be due to differences in HF etiology, with the German studies not specifying etiology, but the UK and Ireland study including cases mainly due to heart muscle diseases. However, even within the UK and Ireland, the incidence varied, with rates from Ireland and Scotland ranging from 0.11 to 1.27 per 100,000, respectively [13].

A wider variation was observed in Nigerian studies, which showed HF prevalence ranging from 2.7 to 9% in children presenting to the emergency room or admitted into pediatric wards [18, 19, 22, 23]. The differences in HF prevalence from different Nigerian centers could be due to differences in the study designs, patient selection, diagnosis and definition of HF, and the different time periods in which the studies were conducted. Similar differences in diagnosis and definition may underlie the differences in the rate of HF prevalence associated with RF reported in two Turkish studies (9% [54] and 1.5% [53]).

Overall, comparisons between studies and countries need to be interpreted with caution as the studies were highly heterogeneous and reported diverse etiologies across countries.

Leading causes of pediatric HF reported from lower income countries were lower respiratory tract infections and severe anemia [18, 19, 22, 23]. Inadequate treatment for conditions such as malaria, which can cause severe anemia and associated HF, may be a reason for the above finding [18, 19, 22, 23]. In comparison, studies from the developed world reported congenital heart disease and cardiomyopathies as two leading causes of HF in the pediatric population, with other major causes including rhythm and conduction disturbances and acquired heart diseases [13, 17].

More than half of the studies included in the review summarized evidence of HF incidence/prevalence diagnosed secondary to another CVD. Only three studies on the incidence of HF secondary to CHD were identified in this review, including two studies with rare etiology (secondary to Scimitar syndrome [25] and trisomy 21 with congenital heart disease [26]). It is widely recognized that many infants with left heart obstructive lesions and large VSDs will present with HF [89], but data on the incidence are lacking. Most reports of HF prevalence were in the context of congenital heart disease, particularly VSD and ASD [31–34, 36, 90]. Similarly, this is likely due to a reporting bias, as some of the other congenital heart diseases that are associated with HF may be under-reported.

A high HF prevalence was observed when congenital heart disease co-existed with conditions such as malnutrition, pneumonia, and trisomy 21 [26, 35, 37, 39, 91]. Findings from these studies also suggest that spontaneous closure of ASDs/VSDs was less common in young children with co-existing HF than in those without HF [33, 36, 55].

Evidence suggests that approximately 40% of children with symptomatic cardiomyopathy develop HF of such severity that it leads to transplantation or death [92]. This review provides information on the incidence and prevalence of HF in different types of cardiomyopathies, including DCM, HCM, and RCM and myocarditis [13, 40, 44–49]. We found that the proportion of HF was highest among patients with DCM, followed by patients with RCM and then HCM [13, 40, 44–46, 48, 49]. Additionally, we found that HF is a major complication in conditions such as acute rheumatic fever, rheumatic heart disease, and IE [30, 53, 56].

The third disease category summarized evidence of pediatric HF incidence/prevalence diagnosed secondary to non-CVDs. Anthracyclines are used widely for the treatment of numerous childhood malignancies and have known cardiac toxicity. The data indicate that the risk of developing HF is related to the treatment dose or mode of delivery (pulsatile versus continuous). Many patients developed HF within the first year of treatment [64–66], and that younger children were more vulnerable to anthracycline cardiotoxicity [64–66].

The close relationship between HF and renal disorders is reflected in our findings. The studies on renal disorders included patients with AKI, acute renal failure, or with AGN due to PIGN. While HF was a presenting symptom in patients with PIGN, it was reported as an etiology for AKI or acute renal failure, along with other conditions [72–78, 86, 87]. Another major area in which HF was reported was among pediatric HIV/AIDS patients. The studies reported a wide range of prevalence from different geographic locations owing to the fact that the included patients were in different stages of HIV, across different pediatric ages, and it was noted that the rate of cardiac complications increases as these patients progress to AIDS [67, 79–81, 93, 94].

### Limitations

In all three disease categories, a lack of large population-based studies and the heterogeneity of study design limit the scope for generalizations and comparisons. Therefore, differences between studies and countries need to be interpreted with caution. Furthermore, much of the evidence was derived from hospital-based studies, introducing a greater potential for selection bias compared with population-based studies.

The large proportion of full-text studies (63 of 77) that were graded as ‘poor’ according to the Downs and Black checklist suggests the need for studies with improved design and methodology. Furthermore, the development of standardized definitions of pediatric HF would help in reducing heterogeneity, facilitating higher quality comparisons of outcomes between studies.

The search strategy did not include the various comorbid conditions as dedicated search terms. Therefore, relevant articles could have been missed. Nevertheless, we believe that the comprehensive nature of our methodology ensured that the prevalence/incidence of HF in all major CVDs and non-CVDs in the pediatric population is captured.

## Conclusion

In summary, this systematic review provides valuable information and insights into the incidence and prevalence of HF in children and adolescents over the last 20 years (1996–2016) and strengthens the current knowledge on the epidemiology of pediatric HF. While a substantial number of studies were identified, more large population-based studies are needed to consolidate the evidence base. Moreover, there is a need to use standard definitions for HF in future pediatric epidemiological studies, to assess the true differences in incidence and prevalence among various studies.

## Electronic supplementary material

Below is the link to the electronic supplementary material.

Supplementary material 1 (DOC 927 KB)
