# Supplementary material for: Systematic Literature Review on the Incidence and Prevalence of Heart Failure in Children and Adolescents
Source: Pediatr Cardiol. 2017 Dec 20;39(3):415–36. doi: 10.1007/s00246-017-1787-2 (PMC5829104; doi:10.1007/s00246-017-1787-2)
Supplement: Supplementary file 1 — Supplementary material 1 (DOC 927 KB) [file 246_2017_1787_MOESM1_ESM.doc]

**Supplementary Appendix**

**Systematic literature review on the incidence and prevalence of heart failure in children and adolescents**

Robert E Shaddy,^1^ Aneesh Thomas George,^2^ Thomas Jaecklin,^3^ Eimear Nic Lochlainn,^4^ Lalit Thakur,^2^ Rumjhum Agrawal,^2^ Susan Solar-Yohay,^5^ Fabian Chen,^5^ Joseph W Rossano,^6^ Thomas Severin,^4^ Michael Burch^6^

^1^Children's Hospital Los Angeles, Los Angeles, California, United States; ^2^Novartis Healthcare Pvt. Ltd., Hyderabad, India; ^3^Shire International GmbH, Global Clinical Development, Zählerweg 10, 6300 Zug, Switzerland; ^4^Novartis Pharma AG, Basel, Switzerland; ^5^Novartis Pharmaceuticals Corporation, East Hanover, NJ, USA; ^6^Children's Hospital of Philadelphia, University of Pennsylvania Perelman School of Medicine, Philadelphia, Pennsylvania, USA; ^7^Cardiorespiratory Division, Great Ormond Street Hospital for Children, London, UK

Contents

[Systematic search strategy 2](#__RefHeading___Toc468449872)

[Data from all included studies 5](#__RefHeading___Toc468449873)

[A. Studies on primary HF diagnosis 5](#__RefHeading___Toc468449874)

[B. Studies on secondary HF diagnosis In CVDs 19](#__RefHeading___Toc468449875)

[C. Studies on secondary HF diagnosis In non-CVDs 36](#__RefHeading___Toc468449876)

[References 46](#__RefHeading___Toc468449877)

# Systematic search strategy

| **S. No** | **Parameter** | **String** | **No. of hits** |
| --- | --- | --- | --- |
| **1** | Disease term | exp heart failure/ | 353,480 |
| **2** |  | ((heart or cardiac or myocard*) adj2 (fail* or insufficien* or decomp*)).tw. | 275,515 |
| **3** |  | 1 or 2 | 433,280 |
| **4** | Population | exp Child/ | 2,113,965 |
| **5** |  | exp Adolescent/ not exp Adult/ | 577,741 |
| **6** |  | exp Pediatrics/ | 86,661 |
| **7** |  | exp Infant/ | 939,756 |
| **8** |  | (Pediatric* or paediatric* or neonat* or perinat* or child* or juvenile* or bab*3 or infant* or toddler* or newborn or new-born or premature* or preterm* or pre-term* preschool* or pre-school* or teen* or adolescen* or minor* or pubescen*).tw. | 3,076,866 |
| **9** |  | 4 or 5 or 6 or 7 or 8 | 3,932,775 |
| **10** | Outcomes | (prevalen* or inciden*).mp. | 2,381,389 |
| **11** | Combining Disease, Population and Outcomes | 3 and 9 and 10 | 6,502 |
| **12** | Limiting with Study Types | exp case report/ or exp case study/ or exp case series/ or exp abstract report/ or exp editorial/ or exp letter/ or exp news/ | 4,002,644 |
| **13** |  | 11 Not 12 | 5,923 |
| **14** | Additional Limits | Limit 13 with “ English Language” , “Human”, “Humans” and Years “(1996 to Current)” | 4,970 |
| **15** | Remove Duplicate Articles |  | 4,150 |
| **16** | Embase Limits for Age (Not valid in Medline) | limit 18 to (infant <to one year> or child <unspecified age> or preschool child <1 to 6 years> or school child <7 to 12 years> or adolescent <13 to 17 years>) | 2,013 |
| **17** | Medline Limits for Age, applied to articles from step16 (Not valid in Embase) | limit 19 to ("all infant (birth to 23 months)" or "all child (0 to 18 years)" or "newborn infant (birth to 1 month)" or "infant (1 to 23 months)" or "preschool child (2 to 5 years)" or "child (6 to 12 years)" or "adolescent (13 to 18 years)") | 1,952 |

**Systematic review summary protocol**

| **Objective** | |
| --- | --- |
| Primary study question/objective | The aim of this systematic review was to perform a narrative review of the evidence on the incidence and prevalence of HF in children and adolescents (birth to <18 years of age) |
| **Studies to include** | |
| Study design | Observational studies |
| Population | Neonates, infants, children, and adolescents (birth to <18 years of age) having HF, AHF, CHF, or congestive heart failure |
| Interventions and Comparator | N/A |
| Outcomes | Incidence and /or prevalence of HF, and distribution of HF in various subgroups of the population contributing to incidence/ prevalence data (<18 years) |
| Other specific inclusion/exclusion criteria | Study duration and country: No limits  Sample size: Studies with a sample size of less than 10 patients were excluded  Studies without age-specific data or those that did not specify the age range of interest were excluded  Studies that have no information on the base population (denominator) were excluded and in such cases no secondary sources were used to get this information |
| **Publication information** | |
| Language and search timeframe | English language, 1996 to February 04, 2016 |
| Databases | Embase 1996 to 2016 February 04  Ovid MEDLINE(R) without revisions 1996 to January week 4 2016  Ovid MEDLINE(R) In-process & other non-Indexed citations February 04, 2016 |
| Grey Literature | Conference proceedings (2012 to 2015)   - ISHLT ,World Congress Pediatric Cardiology & Cardiac Surgery ,AHA, AEPC, Society of Pediatric Research annual meeting and European Society of Pediatric Research annual meeting   Bibliographic search of included studies  Other sources: NHS EED, HTA, and WHO |
| **Overview of Information extracted** | |
| Study information | Individual study (a combination of the first author’s last name and the publication year),  year of publication, authors, title of study, full text/conference abstract, study objective, study design, country/region, number of centers, setting, population studied, inclusion/exclusion criteria, total population before study initiation/at the beginning of the study, timescale, follow-up period, type of HF reported (AHF/CHF/HF/congestive HF), and type of outcomes reported (incidence/prevalence) |
| Baseline data | Defining the study group, study group sample size, age, gender, race |
| Data to be extracted | Data on incidence and/or prevalence of HF (AHF/CHF/HF/congestive HF)  Data on HF (AHF/CHF/HF/congestive HF) as a comorbid condition (if available)*  Data on the distribution of HF in various sub-groups of the HF population |
| Down’s and Black critical appraisal checklist | Reporting external validity, internal validity (bias), internal validity - confounding (selection bias), and power of all full-text articles ( not conference abstracts) |
| Abbreviations: AEPC, Association for European Paediatric and Congenital Cardiology; AHA, American Heart Association; AHF, acute heart failure; CHF, chronic heart failure; HF, heart failure; HTA, Health Technology Assessment; ISHLT, The International Society for Heart & Lung Transplantation; N/A, not applicable; NHS EED, National Health Service Economic Evaluation Database; WHO, World Health Organization  *The outcome was only perused if the abstract of the study indicated (in the first phase of screening) that HF was present as a comorbid condition in patients with other disease. This is because HF as a comorbid condition was not factored in the search strategy. Also, HF can be reported as a comorbid condition in any disease, without any hint regarding the same in the abstracts. | |

# Data from all included studies

## Studies on primary HF diagnosis

**Table A1**: Incidence of HF in all studies on primary HF diagnosis

| **Study name** | **Study design** | **Country, Period** | **Setting** | **Study population, (age range)** | **Subgroups** | **Type of HF** | **Sample size** | **Gender** | | **HF incidence and distribution in study sub-groups** | | | |
| --- | --- | --- | --- | --- | --- | --- | --- | --- | --- | --- | --- | --- | --- |
|  |  |  |  |  |  |  |  | **Female (n)** | **Female (%)** | **Cases (n)** | **Incidence (%)** | **Incidence per 100,000** | **Distribution of incident cases of HF in study subgroups (%)** |
| Massin 2008[1] | Prospective | Belgium, 1996 to 2006 (10 years) | Hospital (1) | Hospitalized cases (0 to 16 years) | All patients | HF | 1,196 | 620 | 51.8* | 124 | 10.4 | - |  |
|  |  |  |  |  | Infants | HF | 124 | NR | NR | 72 | - | - | 58.1 |
|  |  |  |  |  | NYHA II | HF | 124 | NR | NR | 60 | - | - | 48.4* |
|  |  |  |  |  | NYHA III | HF | 124 | NR | NR | 49 | - | - | 39.5* |
|  |  |  |  |  | NYHA IV | HF | 124 | NR | NR | 15 | - | - | 12.1* |
|  |  |  |  |  | Congenital heart disease (as etiology) | HF | 124 | NR | NR | 64 | - | - | 51.6* |
|  |  |  |  |  | Acquired heart disease (as etiology) | HF | 124 | NR | NR | 23 | - | - | 18.5* |
|  |  |  |  |  | Rhythm and conduction disturbances (as etiology) | HF | 124 | NR | NR | 13 | - | - | 10.5* |
|  |  |  |  |  | Cardiomyopathies  (as etiology) | HF | 124 | NR | NR | 24 | - | - | 19.4* |
| Andrews 2008[2] | Prospective | UK and Ireland, 2003 (1 year) | Hospitals (17) | Hospitalized HF cases (0 to 16 years) | All patients | HF | 11,712,100* | NR | NR | 104 | NR | 0.87 |  |
|  |  |  |  |  | NYHA (III-IV) | HF | 104 | NR | NR | 85* |  |  | 82 |
|  |  |  |  |  | Dilated cardiomyopathy; both familial and idiopathic (as etiology) | HF | 104 | NR | NR | 58 | - | - | 55.8* |
|  |  |  |  |  | Probable myocarditis; including virus confirmed (as etiology) | HF | 104 | NR | NR | 23 | - | - | 22.1* |
|  |  |  |  |  | Occult arrhythmia (as etiology) | HF | 104 | NR | NR | 7 | - | - | 6.7* |
|  |  |  |  |  | Anthracycline toxicity (as etiology) | HF | 104 | NR | NR | 5 | - | - | 4.9* |
|  |  |  |  |  | Metabolic disease (as etiology) | HF | 104 | NR | NR | 4 | - | - | 3.9* |
|  |  |  |  |  | Left ventricular non compaction (as etiology) | HF | 104 | NR | NR | 3 | - | - | 2.9* |
|  |  |  |  |  | Duchenne muscular dystrophy (as etiology) | HF | 104 | NR | NR | 2 | - | - | 2.0* |
|  |  |  |  |  | Restrictive cardiomyopathy | HF | 104 | NR | NR | 1 | - | - | 1.0* |
|  |  |  |  |  | Rickets (as etiology) | HF | 104 | NR | NR | 1 | - | - | 1.0* |
|  |  |  |  |  | Northern England | HF | 2, 847,400 | NR | NR | 28 | NR | 0.98 | - |
|  |  |  |  |  | The Midlands | HF | 1, 911,700 | NR | NR | 24 | NR | 1.25 | - |
|  |  |  |  |  | Southern England | HF | 5, 044 ,400 | NR | NR | 36 | NR | 0.71 | - |
|  |  |  |  |  | Wales | HF | 577, 300 | NR | NR | 1 | NR | 0.17 | - |
|  |  |  |  |  | Scotland | HF | 943 ,100 | NR | NR | 12 | NR | 1.27 | - |
|  |  |  |  |  | Northern Ireland | HF | 388, 200 | NR | NR | 2 | NR | 0.51 | - |
|  |  |  |  |  | Ireland | HF | 895 ,160 | NR | NR | 1 | NR | 0.11 | - |
| Neumann 2009[3] | Retrospective | Germany, 2000 to 2006  (7 years) | Country wide hospitals | Hospitalized HF cases (0 to < 15 years) | Years 2000 to 2006 (7 years) | HF | NR | NR | NR | NR | NR | 2 to 3 | - |
|  |  |  |  |  | Year 2000 | HF | NR | NR | NR | NR | NR | 2 | - |
|  |  |  |  |  | Year 2001 | HF | NR | NR | NR | NR | NR | 3 | - |
|  |  |  |  |  | Year 2002 | HF | NR | NR | NR | NR | NR | 3 | - |
|  |  |  |  |  | Year 2003 | HF | NR | NR | NR | NR | NR | 2 | - |
|  |  |  |  |  | Year 2004 | HF | NR | NR | NR | NR | NR | 2 | - |
|  |  |  |  |  | Year 2005 | HF | NR | NR | NR | NR | NR | 2 | - |
|  |  |  |  |  | Year 2006 | HF | NR | NR | NR | NR | NR | 2 | - |
| Schmidt 2013[4] | Retrospective | Germany, 1995 and 2009 (two distinct years) | Country wide hospitals | Hospitalized HF cases (0 to 14 years) | Year 1995 | HF | 13,238,000 | NR | NR | 265 | NR | 2 | - |
|  |  |  |  |  | Year 2009 | HF | 11,030,000 | NR | NR | 221 | NR | 2 | - |
| Tseng 2010[5] | Retrospective | Taiwan, 2005 (1 year) | Country wide hospitals | Hospitalized HF cases (0 to 14 years) | All patients | HF | 190,374* | 90,873 | 47.7 | 14* | NR | 7.4* | - |
|  |  |  |  |  | 0 to 4 years | HF | 55,274 | 26,319 | 47.6 | 12 | NR | 21.7 | - |
|  |  |  |  |  | 5 to 9 years | HF | 65,636 | 31,355 | 47.8 | 0 | NR | 0 | - |
|  |  |  |  |  | 10 to 14 years | HF | 69,464 | 33,199 | 47.8 | 2 | NR | 2.9 | - |
|  |  |  |  | Males | 0 to 14 years | HF | 99,489* | - |  | 6 | NR | 6* | - |
|  |  |  |  | Females | 0 to 14 years | HF | 90,873* | 90,873 | 100 | 8* | NR | 8.8* | - |
| Abbreviations: HF, heart failure; NR, not reported; NYHA, New York Heart Association.*Calculated values from the source article. | | | | | | | | | | | | | |

**Table A2: Prevalence of HF in all studies on primary HF diagnosis**

| **Study name** | **Study design** | **Country, Period** | **Setting** | **Study population, (age range)** | **Subgroups** | **Type of HF** | **Sample size** | **Gender** | | **HF prevalence and distribution in study sub-groups** | | | |
| --- | --- | --- | --- | --- | --- | --- | --- | --- | --- | --- | --- | --- | --- |
|  |  |  |  |  |  |  |  | **Female (n)** | **Female (%)** | **Cases (n)** | **Prevalence (%)** | **Prevalence per 100,000** | **Distribution of prevalent cases of HF in study subgroups (%)** |
| Jiménez-García 2013 [6] | Cross-sectional | Spain, 2012 to 2013 (1 year) | Community (Madrid) | Influenza vaccination coverage (6 months to 14 years) |  | HF | 981,855 | 477,928 | 48.7 | 818 | 0.1 | 83.3* | - |
| Rodríguez-Rieiro 2010 [7] |  | Spain, 2009 (Point prevalence) |  |  | Patients with chronic diseases | HF | 117,940 | 48,806 | 41.4 | 689 | 0.6* | 7.7^$^ | - |
| Lagunju 2003[8] | Prospective | Nigeria, 2000 to 2001 (10 months) | Hospital (1) | Hospital admissions, (8 days to 12 years) | All patients | Congestive HF | 1,713 | NR | NR | 100 | 5.8 | - | - |
|  |  |  |  |  | Female | Congestive HF | 100 | 44 | 44 | 44 |  | - | 44 |
|  |  |  |  |  | Neonates with HF | Congestive HF | 100 | - | - | 2 | - | - | 2 |
|  |  |  |  |  | Children(1 to 12 months) with HF | Congestive HF | 100 | - | - | 54 | - | - | 54 |
|  |  |  |  |  | Children(1 to 5 years) with HF | Congestive HF | 100 | - | - | 28 | - | - | 28 |
|  |  |  |  |  | Children aged > 5 years of age | Congestive HF | 100 | - | - | 16 | - | - | 16 |
| Adekanmbi 2006[9] | Prospective | Nigeria, 2002 to 2003 (1 year) | Hospital (1) | Hospital admissions and ER (1 day to 14 years) | All patients | Congestive HF | 1,552 | NR | NR | 109^#^ | 7 | - |  |
|  |  |  |  |  | Female | Congestive HF | 104 | 53 | 51 | 53 |  | - | 51 |
|  |  |  |  |  | Children (aged <28 days) | Congestive HF | 104 | NR | NR | 10 | - | - | 9.6 |
|  |  |  |  |  | Children (aged >28 days to 1 year) with HF | Congestive HF | 104 | NR | NR | 51 | - | - | 49.0 |
|  |  |  |  |  | Children (aged >12 months to 5 years) with HF | Congestive HF | 104 | NR | NR | 31 | - | - | 29.8 |
|  |  |  |  |  | Children (aged > 5 to 10 years) with HF | Congestive HF | 104 | NR | NR | 6 | - | - | 5.8 |
|  |  |  |  |  | Children (aged > 10 years) with HF | Congestive HF | 104 | NR | NR | 6 | - | - | 5.8 |
|  |  |  |  |  | Severe anemia alone (as etiology) | Congestive HF | 104 |  |  | 48 |  |  | 46 |
|  |  |  |  |  | LRTI alone ( as etiology) | Congestive HF | 104 |  |  | 30 |  |  | 29 |
|  |  |  |  |  | Anemia with LRTI (as etiology) | Congestive HF | 104 |  |  | 12 |  |  | 11.5 |
|  |  |  |  |  | Congenital heart disease(as etiology) | Congestive HF | 104 |  |  | 11 |  |  | 10.5 |
|  |  |  |  |  | RHD (as etiology) | Congestive HF | 104 |  |  | 1 |  |  | 1 |
|  |  |  |  |  | Myocarditis (as etiology) | Congestive HF | 104 |  |  | 1 |  |  | 1 |
|  |  |  |  |  | Chronic renal disease | Congestive HF | 104 |  |  | 1 |  |  | 1 |
| Oyedeji 2010[10] | Prospective | Nigeria  2007 (6 months) | Hospital (1) | Patients in ER (1 month to 12 years) | All patients | Congestive HF | 391 | NR | NR | 35 | 9 | - | - |
|  |  |  |  |  | Female | Congestive HF | 35 | 14 | 40 | 14 |  |  | 40 |
|  |  |  |  |  | Children (aged 1to 12 months) with HF | Congestive HF | 35 | NR | NR | 13 | - | - | 37.1 |
|  |  |  |  |  | Children (aged >1to 5 years) with HF | Congestive HF | 35 | NR | NR | 15 | - | - | 42.9 |
|  |  |  |  |  | Children (aged >5 to 12 years) with HF | Congestive HF | 35 | NR | NR | 7 | - | - | 20 |
|  |  |  |  |  | Anemia (as an etiology for HF) | Congestive HF | 35 | NR | NR | 20 | - | - | 57.1 |
|  |  |  |  |  | Bronchopneumonia (as an etiology for HF) | Congestive HF | 35 | NR | NR | 10 | - | - | 28.6 |
|  |  |  |  |  | Lobar pneumonia (as an etiology for HF) | Congestive HF | 35 | NR | NR | 3 | - | - | 8.6 |
|  |  |  |  |  | VSD (as an etiology for HF) | Congestive HF | 35 | NR | NR | 1 | - | - | 2.9 |
|  |  |  |  |  | TGA (as an etiology for HF) | Congestive HF | 35 | NR | NR | 1 | - | - | 2.9 |
|  |  |  |  |  | RF (as an etiology for HF) | Congestive HF | 35 | NR | NR | 1 | - | - | 2.9 |
|  |  |  |  |  | Hepatitis induced myocarditis (as an etiology for HF) | Congestive HF | 35 | NR | NR | 1 | - | - | 2.9 |
|  |  |  |  |  | Septicemia (as an etiology for HF) | Congestive HF | 35 | NR | NR | 1 | - | - | 2.9 |
|  |  |  |  |  | Acute glomerulonephritis (as an etiology for HF) | Congestive HF | 35 | NR | NR | 1 | - | - | 2.9 |
| Animasahun 2015[11] | Prospective | Nigeria, 2011 to 2012 (2 years) | Hospital (1) | (1 day to 12 years) | All patients | Congestive HF | 5,705 | NR | NR | 156 | 2.7 | - | - |
|  |  |  |  |  | Female | Congestive HF | 156 | 74 | 47.4 | 74 |  |  | 47.4 |
|  |  |  |  |  | Neonates | Congestive HF | 156 | NR | NR | 5 | - | - | 3.2 |
|  |  |  |  |  | Children aged 1 to 12 months | Congestive HF | 156 | NR | NR | 93 | - | - | 59.6 |
|  |  |  |  |  | Children aged 12 to 60 months | Congestive HF | 156 | NR | NR | 43 | - | - | 27.5 |
|  |  |  |  |  | Children aged > 60 months of age | Congestive HF | 156 | NR | NR | 15 | - | - | 9.6 |
| Abbreviations: ER, emergency room; HF, heart failure; LRTI, lower respiratory tract infection; NR, not reported; RHD, rheumatic heart disease; RF, Rheumatic fever; TGA, transposition of the great arteries; VSD, ventricular septal defect. ^#^ Total HF was 109 patients, data available for only 104 patients. *Calculated values from the source article, ^$^ Prevalence was for per 10,000 inhabitants | | | | | | | | | | | | | |

**Table A3: Age wise distribution of HF prevalence attributed to different etiologies**

| **Study name** | **Study group/ subgroups** | **Type of HF** | **Sample size in the subgroup/ group** | **Etiologies for HF** | **Distribution of prevalent cases of HF in study subgroups** | |
| --- | --- | --- | --- | --- | --- | --- |
|  |  |  |  |  | **Cases (n)** | **Cases (%)** |
| Lagunju 2003[8] | Neonates | Congestive HF | 2 | Severe anemia | 1 | 50 |
|  |  |  |  | Congenital heart disease | 1 | 50 |
|  | Children(1 to 12 months) | Congestive HF | 54 | ARI | 26 | 48 |
|  |  |  |  | Severe anemia | 8 | 15 |
|  |  |  |  | Congenital heart disease | 20 | 37 |
|  | Children(1 to 5 years) | Congestive HF | 28 | ARI | 10 | 35.7 |
|  |  |  |  | Severe anemia | 14 | 50 |
|  |  |  |  | Congenital heart disease | 4 | 14.2* |
|  |  |  |  | Septicemia | 1 | 3.6 |
|  | Children aged > 5 years of age | Congestive HF | 16 | Severe anemia | 5 | 31.2 |
|  |  |  |  | Congenital heart disease | 1 | 6.3 |
|  |  |  |  | RHD | 6 | 37.5 |
|  |  |  |  | Renal disorders | 3 | 18.7 |
|  |  |  |  | Septicemia | 1 | 6.3 |
| Adekanmbi 2006[9] | Neonates | Congestive HF | 10 | Anemia | 3 | 30* |
|  |  |  |  | LRTI | 4 | 40* |
|  |  |  |  | Congenital heart disease | 2 | 20* |
|  | Children (aged 1 to 12 months) | Congestive HF | 51 | Anemia | 31 | 60.7* |
|  |  |  |  | LRTI | 25 | 49.0* |
|  |  |  |  | Congenital heart disease | 6 | 11.8* |
|  | Children (aged >1 to 5 years) | Congestive HF | 31 | Anemia | 20 | 64.5* |
|  |  |  |  | LRTI | 8 | 25.8* |
|  |  |  |  | Congenital heart disease | 2 | 6.5* |
|  | Children (aged > 5 to 10 years) | Congestive HF | 6 | Anemia | 2 | 33.3* |
|  |  |  |  | LRTI | 4 | 66.6* |
|  |  |  |  | Congenital heart disease | 1 | 16.6* |
|  |  |  |  | RHD | 1 | 16.6* |
|  |  |  |  | Myocarditis | 1 | 16.6* |
|  | Children (aged > 10 years) | Congestive HF | 6 | Anemia | 4 | 66.6* |
|  |  |  |  | LRTI | 1 | 16.6* |
|  |  |  |  | Chronic renal disease | 1 | 16.6* |
| Animasahun 2015[11] | Neonates | Congestive HF | 5 | Congenital heart disease | 2 | 40* |
|  |  |  |  | Severe anemia | 3 | 60* |
|  | Children aged 1 to 12 months | Congestive HF | 93 | Acute LRTI | 33 | 35.4* |
|  |  |  |  | Congenital heart defects | 35 | 37.6* |
|  |  |  |  | Severe anemia | 19 | 20.4* |
|  |  |  |  | Sepsis | 6 | 6.4* |
|  | Children aged >12 to 60 months | Congestive HF | 43 | Acute LRTI | 15 | 34.8* |
|  |  |  |  | Congenital heart disease | 10 | 23.2* |
|  |  |  |  | Severe anemia | 17 | 39.5* |
|  |  |  |  | AHD | 1 | 2.3* |
|  | Children aged > 60 months of age | Congestive HF | 16 | Acute LRTI | 2 | 12.5* |
|  |  |  |  | Congenital heart disease | 2 | 12.5* |
|  |  |  |  | Severe anemia | 6 | 37.5* |
|  |  |  |  | AHD | 4 | 25* |
|  |  |  |  | AGN | 1 | 6.25* |
| Abbreviations: AGN; Acute glomerulonephritis; AHD, Acquired heart disease; ARI, Acute respiratory infections; HF, Heart failure; LRTI, Lower respiratory tract infections; RHD, Rheumatic heart disease. *Calculated values from the source article | | | | | | |

## Studies on secondary HF diagnosis In CVDs

**Table B1: Incidence of HF in all CVD studies**

| **Study name** | **Study design** | **Country, Period** | **Setting** | **Study population, (age range)** | **Subgroups** | **Type of HF** | **Sample size** | **Gender** | | **HF Incidence** | |
| --- | --- | --- | --- | --- | --- | --- | --- | --- | --- | --- | --- |
|  |  |  |  |  |  |  |  | **Female (n)** | **Female (%)** | **Cases (n)** | **Incidence (%)** |
| **Congenital heart defects/ disease** | | | | | | | | | | | |
| Najm 1996[12] | Retrospective | Canada, 1975 to 1985, (21 years) | Hospital (1) | Scimitar syndrome, (1 to 335 days) | - | HF | 19 | 14 | 73.7* | 11 | 57.9* |
| Tomlinson 2010[13]^#^ | Retrospective | Jamaica, 1995 to 2004, (10 years) | Hospital (1) | Trisomy 21 with congenital heart disease and cardiac  lesions, (0 to 12 years) | - | Congestive HF | 46 | NR | NR | 11* | 23.9* |
| Hong 2012 [14] | Retrospective | South Korea, 2000 to 2010, (11 years) | Hospital (1) | TGA, (1 to 108 days) | All patients | HF | 28 | 11 | 39.3 | 5 | 17.9 |
|  |  |  |  |  | With VSD | HF | 12 | 5 | 41.7 | 5 | 41.7 |
| **Vascular malformations** | | | | | | | | | | | |
| Rialon 2015[15] | Retrospective | US, 1995 to 2012, (18 years) | Hospital (1) | Hepatic hemangiomas, (0 to<1 year) | All patients | Congestive HF | 72 | NR | NR | 16* | 22.2* |
|  |  |  |  |  | Patients who underwent initial screening for hemangiomas | Congestive HF | 43 | NR | NR | 2 | 5 |
|  |  |  |  |  | Unscreened patients | Congestive HF | 29 | NR | NR | 14 | 48 |
| **Post OHT** | | | | | | | | | | | |
| Murtuza 2013[16] | Retrospective | UK, 2000 to 2011, (8 years) | Hospital (1) | DCM and RCM , (0.1 to 17.1 years) | All patients | VHF (right) | 159* | 83* | 52.2* | 30* | 18.9 |
|  |  |  |  |  | Patients with DCM | VHF (right) | 136 | 74* | 54.4* | 20 | 14.7 |
|  |  |  |  |  | Patients with RCM | VHF (right) | 23 | 9* | 39.1* | 10 | 43.5 |
| LaPage 2010 [17] | Retrospective | US, 1991 to 2006,  (16 years) | Hospital (1) | Tachyarrhythmia, (0 to 17 years) |  | Acute congestive HF | 19 | NR | NR | 2* | 10.5* |
| **IE** | | | | | | | | | | | |
| Marom 2013[18]^$^ | Retrospective | Israel, 1992 to 2004 (12.5 years) | Hospital (1) | IE, (0 to <18 years) | Children with no predisposing factors for IE | HF | 9 | NR | NR | 7 | 77.8 |
| Abbreviations: DCM, dilated cardiomyopathy HF, heart failure; NR, not reported; OHT, orthotopic heart transplantation; RCM, restrictive cardiomyopathy; VHF, ventricular heart failure.*Calculated from source article ^.#^ In Tomlinson et al 30 of the 76 children had congestive HF at presentation and this is captured in the prevalence section below, in 11 of the remaining 46 children congestive HF developed during the study. A total of 41 patients (30+11) had congestive HF in this study. ^$^ In Marom 9 of a total of 51 patients with IE had no predisposing cardiac anomalies (HF cases are new). Of these 7 cases had HF and have contributed to incidence data whereas, 42 patients had predisposing cardiac anomalies (unclear if HF cases are new) and the data is captured in the prevalence section below | | | | | | | | | | | |

**Table B2: Prevalence of HF in all CVD studies**

| **Study name** | **Study design** | **Country, Period** | **Setting** | **Study population, (age range)** | **Subgroups** | **Type of HF** | **Sample size** | **Gender** | | **HF prevalence and distribution in study sub-groups** | | |  | | | | | | | |
| --- | --- | --- | --- | --- | --- | --- | --- | --- | --- | --- | --- | --- | --- | --- | --- | --- | --- | --- | --- | --- |
|  |  |  |  |  |  |  |  | **Female (n)** | **Female (%)** | **Cases (n)** | **Prevalence (%)** | **Distribution of prevalent cases of HF in study subgroups (%)** |  | | | | | | | |
| **Congenital heart defects/ disease** | | | | | | | | | | | | |  | | | | | | | |
| Meberg 1999[19] | Longitudinal (Prospective and retrospective ) | Norway, 1982 to1996 (15 years) | Hospitals (NR) | Congenital heart disease, (2 weeks to 11 years) | Detected subsequent  to discharge from hospital after birth | Decompensation | 84 | NR | NR | 7 | 8.0 | - |  | | | | | | | |
|  |  |  |  |  | VSD |  | 7 | NR | NR | 4 | - | 57.1* |  | | | | | | | |
|  |  |  |  |  | Atrioventricular septal defect |  | 7 | NR | NR | 1 | - | 14.3* |  | | | | | | | |
|  |  |  |  |  | Coarctation of the aorta |  | 7 | NR | NR | 1 | - | 14.3* |  | | | | | | | |
|  |  |  |  |  | Endocardial  fibroelastosis |  | 7 | NR | NR | 1 | - | 14.3* |  | | | | | | | |
| Vaidyanathan 2006[20] | Prospective | India,2005 to 2006 (1 year) | Hospital (1) | Malnutrition with congenital heart disease, (0 to <5 years) |  | Congestive HF | 476 | 243* | 51.2* | 194 | 40.8 | - |  | | | | | | | |
| Okoromah 2011[21] | Case-control | Nigeria,2006 to 2008 (2 years) | Cases; Hospital (1) | Cases: Malnutrition and congenital heart disease  (3 to 192 months) | All cases | Congestive HF | 73 | NR | NR | 60 | 82.2 | - |  | | | | | | | |
|  |  |  |  |  | Ross score (0 to 6) | Congestive HF | 60 | - | - | 16 |  | 26.7* |  | | | | | | | |
|  |  |  |  |  | Ross score (7 to 12) | Congestive HF | 60 | - | - | 44 |  | 73.3 |  | | | | | | | |
|  |  |  | Controls; Community (Primary school) | Controls: Malnutrition with no congenital heart disease (3 to 192 months) | All controls | Congestive HF | 76 | NR | NR | 0 | 0 | - |  | | | | | | | |
| Sadoh 2013[22] | Prospective | Nigeria,2011 to 2012 (1 year) | Hospital (1) | Pneumonia with and without congenital heart disease (1 to 48 months) | All patients | Congestive HF | 121 | 60 | 49.6 | 49 | 40.5 | - |  | | | | | | | |
|  |  |  |  |  | Pneumonia and congenital heart disease | Congestive HF | 14 |  |  | 9 | 64.3 | - |  | | | | | | | |
|  |  |  |  |  | Pneumonia without congenital heart disease | Congestive HF | 107 |  |  | 40 | 37.4 | - |  | | | | | | | |
| Tomlinson 2010[13] | Retrospective | Jamaica,1995 to 2004 (10 years) | Hospital (1) | Trisomy 21 with congenital heart disease, (0 to 12 years) | - | Congestive HF | 76 | 46 | 60 | 30 | 39.5* | - |  | | | | | | | |
| Shah 2008[23] | Retrospective | Nepal,2006 (1 year) | Hospital (1) | Congenital heart disease, (0 to <15 years) | - | Congestive HF | 84 | 33 | 39.3 | 46 | 54.8 | - |  | | | | | | | |
| Miyake 2004 [24] | Prospective | Japan, 1986 to 1996 (11 years) | Hospital (1) | VSD, (1 to 88 days) | All patients | Congestive HF | 225 | 109 | 48.4* | 104 | 46.0 | - |  | | | | | | | |
|  |  |  |  |  | Subpulmonary VSD | Congestive HF | 104 | NR | NR | 18* | - | 17.3* |  | | | | | | | |
|  |  |  |  |  | Perimembranous VSD | Congestive HF | 104 | NR | NR | 85 | - | 81.7* |  | | | | | | | |
|  |  |  |  |  | Muscular | Congestive HF | 104 | NR | NR | 1 | - | 1* |  | | | | | | | |
|  |  |  |  |  | Spontaneous closure | Congestive HF | 104 | NR | NR | 20 | - | 19.2* |  | | | | | | | |
|  |  |  |  |  | Small open | Congestive HF | 104 | NR | NR | 31 | - | 29.8* |  | | | | | | | |
|  |  |  |  |  | Surgical closure | Congestive HF | 104 | NR | NR | 53 | - | 51* |  | | | | | | | |
| Sadoh 2010[25] | Prospective | Nigeria, 2006 to 2009 (2 years and 5 months) | Hospital (1) | VSD, (2 to 24 months) | All patients | Congestive HF | 61 | 35 | 57.4 | 15 | 24.6 | - |  | | | | | | | |
|  |  |  |  |  | Spontaneous closure | Congestive HF | 15 | NR | NR | 3* | - | 20 |  | | | | | | | |
| Najm 1998[26] | Retrospective | Canada, 1982 to 1996 (14 years and 5 months) | Hospital (1) | ASD, (1 month to 16.4 years) | - | Congestive HF | 180 | 97 | 53.9* | 35 | 20 | - |  | | | | | | | |
| Azhari 2004[27] | Retrospective | Saudi Arabia, 1990 to 2003 (14 years and 1 month) | Hospital (1) | ASD, (1 day to 11 years) | All patients | Congestive HF | 121 | 74 | 61.2 | 14 | 11.6 | - |  | | | | | | | |
|  |  |  |  |  | Small defects | Congestive HF | 22 | 9* | 41 | 0 | 0 | - |  | | | | | | | |
|  |  |  |  |  | Medium defects | Congestive HF | 27 | NR | NR | 1 | 3.7* | - |  | | | | | | | |
|  |  |  |  |  | Large defects | Congestive HF | 72 | NR | NR | 13 | 18.1* | - |  | | | | | | | |
|  |  |  |  |  | Pulmonary arterial hypertension@ | Congestive HF | 8 | 2 | 25 | 8 | 100* | - |  | | | | | | | |
| Trivedi 2006[28] | Retrospective | Canada, 1970 to 2001 (31 years and 4 months) | Hospital (1) | Ductal origin of the distal pulmonary artery, (0 to 6.5 years) | - | Congestive HF | 45 | NR | NR | 12 | 26.7* | - |  | | | | | | | |
| Harshangi 2013[29] | Prospective | India,2005 to 2007 (1 year) | Hospital (1) | Congenital heart disease, (1 month to 1 year) | - | Congestive HF | 50 | NR | NR | 28 | 56 | - |  | | | | | | | |
| Agny 1999[30] | Retrospective | US,1981 to 1997 (16 years) | Hospital (1) | ASD (3 to 58 months) | - | Congestive HF | 38 | 17 | 44.7* | 16* | 41 | - |  | | | | | | | |
| Yim 2015[31] | Retrospective | Australia, 1982 to 2012 (30 years and 4 months) | Hospital (1) | CAF, (3 days to 5.2 years) | - | Congestive HF | 13 | 9 | 69.2 | 4 | 30.8 | - |  | | | | | | | |
| Mottin 2013[32] | Retrospective | France,1990 to 2001 (11 years) | Hospital (1) | CAF, (0 to 15.4 years) | - | Congestive HF | 61 | 36 | 59.0 | 7* | 11 | - |  | | | | | | | |
| Parvathy 2000[33] | Prospective | India, (5 years) | Hospital (1) | Down’s syndrome with congenital heart defects, (1 month to 14 years) | - | Congestive HF | 21 | 12 | 57.2* | 5 | 23.8* | - |  | | | | | | | |
| Adeeb 2004[34] | Retrospective | Malaysia, 1983 to 1994 (11 years and 9 months) | Hospital (1) | Coarctation of the aorta, (0 to 9 years) | All patients | HF | 101* | NR | NR | 50* | 49.5* | - |  | | | | | | | |
|  |  |  |  |  | 0 to 29 days of age | HF | 14 | NR | NR | 8 | 57.1* | - |  | | | | | | | |
|  |  |  |  |  | 1 to 11 months of age | HF | 42 | NR | NR | 42 | 100* | - |  | | | | | | | |
|  |  |  |  |  | 1 to 4 years | HF | 33 | NR | NR | 0 | 0 | - |  | | | | | | | |
|  |  |  |  |  | 5 to 9 years | HF | 12 | NR | NR | 0 | 0 | - |  | | | | | | | |
| **Cardiomyopathies** | | | | | | | | | | | | |  | | | | | | | |
| Skinner 1997[35] | Retrospective | England and Wales,1969 to 1994 (25 years) | Hospitals (NR) | Idiopathic (or familial) HCM and/ or Noonan syndrome, (0 to 12 months) | - | HF | 29 | NR | NR | 17 | 59 | - |  | | | | | | | |
| Nugent 2003[36] | Retrospective | Australia,1987 to 1996 (10 years) | Hospitals (21) | Cardiomyopathies, (0 to <10 years) | All patients | Congestive HF | 314 | 148* | 47.1* | 206* | 65.6* | - |  | | | | | | | |
|  |  |  |  |  | DCM | Congestive HF | 184 | 103 | 56 | 165 | 89.7 | - |  | | | | | | | |
|  |  |  |  |  | HCM | Congestive HF | 80 | 25 | 31.2 | 6 | 7.5 | - |  | | | | | | | |
|  |  |  |  |  | RCM | Congestive HF | 8 | 4 | 50 | 4 | 50 | - |  | | | | | | | |
|  |  |  |  |  | Unclassified cardiomyopathy | Congestive HF | 42 | 16 | 38.1 | 31 | 73.8 | - |  | | | | | | | |
| Wilkinson 2015, [37]  Alvarez 2011,[38]  Webber 2012[39] , Colan 2007[40] Towbin 2006 [41], Everitt 2014 [42] (PCMR studies) | Longitudinal (Prospective and Retrospective cohorts) | US, Canada,1990 (ongoing) | Hospitals (98 pediatric cardiac centers for the prospective cohort and 39 tertiary care centers for the retrospective cohort) | Cardiomyopathies , (0 to <18 years) | All patients | Congestive HF | 3,549^±^ | NR | NR | NR | NR | - |  | | | | | | | |
|  |  |  |  |  | All HCM patients | Congestive HF | 849 | NR | NR | 115* | 13.5* | - |  | | | | | | | |
|  |  |  |  |  | Inborn errors of metabolism | Congestive HF | 74 | NR | NR | 30* | 40.3 | - |  | | | | | | | |
|  |  |  |  |  | Malformation syndromes | Congestive HF | 77 | NR | NR | 18* | 23.4 | - |  | | | | | | | |
|  |  |  |  |  | Neuromuscular disorders | Congestive HF | 64 | NR | NR | 4* | 6.4 | - |  | | | | | | | |
|  |  |  |  |  | Infantile/ idiopathic | Congestive HF | 634 | NR | NR | 63* | 9.9 | - |  | | | | | | | |
|  |  | US, Canada, 1990 to 2007 (18 years) |  | DCM, (0 to <18 years) | All DCM patients | Congestive HF | 1,682 | 777* | 46.2* | 1,205* | 71.6* | - |  | | | | | | | |
|  |  |  |  |  | Idiopathic DCM | Congestive HF | 1,192 | 599* | 50.2 | 894 | 75 | - |  | | | | | | | |
|  |  |  |  |  | Neuromuscular Disease | Congestive HF | 139 | 5* | 3.6* | 40 | 28.8 | - |  | | | | | | | |
|  |  |  |  |  | Familial isolated DCM | Congestive HF | 79 | 35* | 44.3* | 44 | 55.7 | - |  | | | | | | | |
|  |  |  |  |  | Myocarditis | Congestive HF | 272 | 138* | 51* | 227 | 83.4 | - |  | | | | | | | |
|  |  | US, Canada,1990 to 2008 (19 years) |  | RCM, (0 to <18 years) | All RCM patients | Congestive HF | 152 | 79* | 52* | 56* | 37 | - |  | | | | | | | |
|  |  |  |  |  | Pure RCM | Congestive HF | 101 | 51* | 51* | 42* | 42 | - |  | | | | | | | |
|  |  |  |  |  | RCM/HCM | Congestive HF | 51 | 27* | 53* | 13* | 26 | - |  | | | | | | | |
| Marcos-Alonso 2013[43] | Retrospective | Spain,2002 to 2012 (11 years) | Hospital (1) | Cardiomyopathy,( 0 to <15 years) | - | HF | 57 | 22 | 38.6 | 25 | 43.8 | - |  | | | | | | | |
| Tsirka 2004[44] | Retrospective | US,1990 to 1999 (10 years) | Hospitals (2) | DCM, (0 to 17.8 years) | - | Congestive HF | 91 | 33* | 36.3* | 72 | 79 | - |  | | | | | | | |
| Soongwang 2002[45] | Retrospective | Thailand, 1996 to 2000 (5 years) | Hospitals (5) | Myocardial diseases, (0.1 to 14.5 years) | All Patients | Congestive HF | 209 | 117* | 56.0 | 151* | 72.0 | - |  | | | | | | | |
|  |  |  |  |  | DCM | Congestive HF | 94 | 51 | 54.3 | 79 | 84.1 | - |  | | | | | | | |
|  |  |  |  |  | Acute myocarditis | Congestive HF | 57 | 38 | 66.7 | 45 | 78.9 | - |  | | | | | | | |
|  |  |  |  |  | HCM | Congestive HF | 38* | 18 | 47.4 | 17 | 44.7 | - |  | | | | | | | |
|  |  |  |  |  | Hypertrophic obstructive cardiomyopathy | Congestive HF | 17* | 8 | 47.1 | 8 | 47.1 | - |  | | | | | | | |
|  |  |  |  |  | RCM | Congestive HF | 3 | 2 | 66.7 | 2 | 66.6 | - |  | | | | | | | |
| Saji 2012[46] | Retrospective | Japan,1997 to 2002 (6 years) | Hospitals (65) | Myocarditis, (1 month to 17 years) | All patients | HF | 169 | NR | NR | 61 | 36.1 | - |  | | | | | | | |
|  |  |  |  |  | Fulminant myocarditis | HF | 64 | NR | NR | 34 | 53.1 | - |  | | | | | | | |
|  |  |  |  |  | Acute myocarditis | HF | 89 | NR | NR | 27 | 30.3 | - |  | | | | | | | |
|  |  |  |  |  | Chronic myocarditis | HF | 8 | NR | NR | NR | NR | - |  | | | | | | | |
|  |  |  |  |  | Myocarditis of unknown type | HF | 8 | NR | NR | NR | NR | - |  | | | | | | | |
| **Rheumatic fever/ Rheumatic heart disease** | | | | | | | | | | | | |  | | | | | | | |
| Bejiqi 2012[47] | Retrospective | Kosovo,1999 to 2010 (11 years) | Hospital (1) | Acute RF, (5 to 17 years) | - | HF | 221 | 114 | 49 | 38 | 17.2* | - |  | | | | | | | |
| Örün 2012[48] | Retrospective | Turkey,1980 to 2009 (30 years) | Hospital (1) | Acute RF (2 to 15 years) | - | HF | 1,115 | 510 | 45.8 | 100 | 9.0* | - |  | | | | | | | |
| Karlassan 2000[49] | Retrospective | Turkey,1993 to 1998 (5 years) | Hospital (1) | Acute RF, (5 to 17 years) | - | Congestive HF | 274 | 147 | 53.6 | 4 | 1.5* | - |  | | | | | | | |
| da Silva 1999[50] | Retrospective | Brazil,1989 to 1994 (6 years) | Hospitals (7) | RF, (3 to 17 years) | - | HF | 786 | 382 | 48.7 | 119 | 15.1* | - |  | | | | | | | |
| Qurashi 2009[51] | Longitudinal  (Retrospective and prospective) | Saudi Arabia,1994 to 2003 (10 years) | Hospital (1) | Acute RF (4 to 12 years) | - | HF | 83 | NR | NR | 14 | 16.9* | - |  | | | | | | | |
| Rayamajhi 2007[52] | Prospective | Nepal, 2003 to 2005 (2 years) | Hospital (1) | Acute RF, (5 to 14 years) | - | HF | 51 | NR | NR | 14 | 28 | - |  | | | | | | | |
| Prakoso 2014[53] | Retrospective | Indonesia,2006 to 2012 (7 years) | Hospital (1) | RHD, (6 to 17 years) | - | Congestive HF | 85 | 45 | 52.9 | 31 | 36.5 | - |  | | | | | | | |
| Thakur 1996[54] | Cross-sectional | India,1992 to 1993 (1 year) | Schools (62) | RHD, (5 to 16 years) |  | Congestive HF | 44 | NR | NR | 2 | 4.5 | - |  | | | | | | | |
|  |  |  |  |  |  | NYHA | 44 | NR | NR | 34 | 77.3 | - |  | | | | | | | |
|  |  |  |  |  |  | NYHA 1 | 34 | - | - | 13 | - | 38.2 |  | | | | | | | |
|  |  |  |  |  |  | NYHA II | 34 | - | - | 13 | - | 38.2 |  | | | | | | | |
|  |  |  |  |  |  | NYHA III | 34 | - | - | 7 | - | 20.7 |  | | | | | | | |
|  |  |  |  |  |  | NYHA IV | 34 | - | - | 1 | - | 2.9 |  | | | | | | | |
| Gapu 2015[55] | Cross-sectional | Zimbabwe, 2012 to 2013 (11 months ) | Hospitals (2) | Acute RF and/or RHD (1 to 12 years) | All patients | Any HF | 50 | 32 | 64.0 | 37* | 74* | - |  | | | | | | | |
|  |  |  |  |  | Outpatients | Chronic HF | 19 | NR | NR | 15 | 78.9 | - |  | | | | | | | |
|  |  |  |  |  | Hospitalized children with Acute RF and/or RHD | Congestive HF | 31 | NR | NR | 22 | 71.0* | - |  | | | | | | | |
|  |  |  |  |  | Hospitalized with RHD only | AHF | 22 | NR | NR | 20 | - | 90.9 |  | | | | | | | |
|  |  |  |  |  | Hospitalized with Acute RF only | AHF | 9 | NR | NR | 2 | - | 22.2 |  | | | | | | | |
| Bitar 2000[56] | Retrospective | Lebanon,1980 to 1995 (16 years) | Hospital (1) | RF, (3 to 17 years) | - | Acute congestive HF | 91 | 38* | 42* | 40* | 44 | - |  | | | | | | | |
| **IE** | | | | | | | | | | | | |  | | | | | | | |
| Lertsapcharoen 2005[57] | Retrospective | Thailand, 1987 to 2004  (18 years) | Hospital (1) | IE, (2 months to 15 years) | - | Congestive HF | 57 | 28 | 49.1* | 15 | 26 | - |  | | | | | | | |
| Marom 2013[18] | Retrospective | Israel, 1992 to 2004 (12 years, 6 months) | Hospital (1) | IE, (0 to <18 years) | Children with predisposing factors for IE ^€^ | HF | 42 | NR | NR | 10 | 23.8 | - |  | | | | | | | |
| Sadiq 2001[58] | Prospective | Pakistan, 1997 to 2000 (4 years) | Hospital (1) | IE (4 months to 16 years) | All patients | HF | 45 | 15 | 33.3* | 18 | 40 | - |  | | | | | | | |
|  |  |  |  |  | Rheumatic heart disease | HF | 24 | - | - | 10 | 42 | - |  | | | | | | | |
|  |  |  |  |  | Congenital heart disease | HF | 20 | - | - | 8 | 40 | - |  | | | | | | | |
|  |  |  |  |  | Myocarditis | HF | 1 | - | - | 0 | 0 |  |  | | | | | | | |
| **Vascular Malformations** | | | | | | | | | | | | |  | | | | | | | |
| Caldarelli 1997[59] | Prospective | Italy, 1980 to 1995 (15 years) | Hospital (1) | Intracranial vascular malformations, (1 to 15 years) | - | Congestive HF | 39 | 14 | 35.9* | 2 | 5.1* | - |  | | | | | | | |
| **Rhythm and conduction disturbances** | | | | | | | | | | | | |  | | | | | | | |
| Massin 2008[60] | Retrospective | Belgium,1995 to 2006 (11 years) | Hospitals (3) | Tachyarrhythmia, (0 to <16 years) | All patients | HF | 250 | 92* | 36.8* | 49 | 19.6* |  |  | | | | | | | |
|  |  |  |  |  | Beginning HF | HF | 49 | - | - | 29 |  | 59.1* |  | | | | | | | |
|  |  |  |  |  | Severe HF | HF | 49 | - | - | 8 |  | 16.3* |  | | | | | | | |
|  |  |  |  |  | Cardiogenic shock | HF | 49 | - | - | 12 |  | 24.5* |  | | | | | | | |
|  |  |  |  |  | Infants | HF | 109 | - | - | 33 | 30.3* |  |  | | | | | | | |
| **Others** | | | | | | | | | | | | |  | | | | | | | |
| Estepa 2001[61] | Prospective | Spain,1977 to 1998 (21 years) | Hospital (1) | RVH, (5 days to 15 years) | - | Congestive HF | 20 | 11 | 55.0* | 6 | 30* | - |  | | | | | | | |
| Jozwiak 2006[62] | Prospective | Poland,1986 to 2004 (18 years) | Hospital (1) | TSC, (0 to 23 years) | CRs (age <2 years) | HF | 42 | 19 | 45.2* | 4 | 9.5* | - |  | | | | | | | |
| Borzouee 2008[63] | Retrospective | Iran,2001 to 2003 (2 years) | Hospital (1) | Cardiac problems, (1 day to 16 years) |  | HF | 1,817 | NR | NR | 25 | 1.4 | - |  | | | | | | | |
| Kayali 2015[64] | Retrospective | Turkey,2005 to 2014 (10.5 years) | Hospital (1) | Cardiac tumours, (1 day to 12 years) | - | HF | 17 | 6 | 35.3* | 1 | 5.9 | - |  | | | | | | | |
| Benzecry 2008[65] | Prospective | Brazil, NR | Hospital (1) | Heart disease, (1 month to 14 years) | All Patients | Congestive HF | 35 | 17* | 48.6* | 8 | 22.9 | - |  | | | | | | | |
|  |  |  |  |  | Complete atrioventricular septal  defect | Congestive HF | 8 | - | - | 3 | - | 37.5 |  | | | | | | | |
|  |  |  |  |  | Hypertrophic myocardiopathy | Congestive HF | 8 | - | - | 4 | - | 50 |  | | | | | | | |
|  |  |  |  |  | Double outlet left ventricle | Congestive HF | 8 | - | - | 1 | - | 12.5 |  | | | | | | | |
| Abbreviations: ASD: Atrial Septal Defects; CAF: Congenital Coronary Artery Fistula; CR: Cardiac rhabdomyomas; DCM: Dilated Cardiomyopathy; HCM: Hypertrophic cardiomyopathy; HF: Heart Failure; IE: Infective endocarditis; NR: Not Reported ; RCM: Restricted Cardiomyopathy; RF: Rheumatic Fever; RHD: Rheumatic Heart Diseases ;RVH: Renovascular Hypertension; TGA: Trans positioning of Great Arteries; TSC: Tuberous Sclerosis Complex; VSD: Ventricular Septal Defect.* Calculated from source article. @ In Azhari 2004 the patients with pulmonary arterial hypertension is inclusive pf patients with small, medium or large defects and so is not a stand-alone group. ±  3,549 is the most recent number of total patients with different cardiomyopathies(HCM, DCM,RCM) from PCMR registry studies. However the total of HCM, DCM and RCM does not add up to this number (Wilkinson 2015). The data for HF in HCM, DCM and RCM are taken from different PCMR publications. ^€^ Data on 9 children without predisposing factors in Marom 2013 are present in the incidence table above, so the total dose not add up to 51 | | | | | | | | | | | | |  |  |  |  |  |  |  | - |

## Studies on secondary HF diagnosis In non-CVDs

**Table C1: Incidence of HF in all non CVD studies**

| **Study name** | **Study design** | **Country, Period** | **Setting** | **Study population, (age range)** | **Subgroups** | **Type of HF** | **Sample size** | **Gender** | | **HF incidence** | | |
| --- | --- | --- | --- | --- | --- | --- | --- | --- | --- | --- | --- | --- |
|  |  |  |  |  |  |  |  | **Female (n)** | **Female (%)** | **Cases (n)** | **Incidence (%)** | |
| **Hematology/ Oncology** | | | | | | | | | | | | |
| van Dalen 2006[66] | Retrospective | Netherlands, 1976 to 2001 (26 years) | Hospital (1) | Anthracyclines for childhood cancer, (<2 to **>**16 years) | Age <2 to 16 years | Congestive HF | 808* | NR | NR | 17* | | 2.1* |
| Berrak 2001[67] | Retrospective | US, 1988 to 1998 (10 years) | Hospital (1) | Doxorubicin for childhood cancer (7 months to 17 years) | - | Congestive HF | 97 | 38 | 39.2* | 1 | | 1.0* |
| Godoy 1997[68] | Retrospective | Japan, 1985 to 1994 (10 years) | Hospital (1) | Anthracyclines for childhood cancer (5 months to 17 years) | - | Congestive HF | 120 | 51 | 42.5* | 6 | | 5.0* |
| **HIV/ AIDS** | | | | | | | | | | | | |
| Starc 2002[69], Fisher 2005[70]  Lipshultz 1998 [71]  Starc 1999[72] | Prospective | US, 1990 to Jan 1997 (6 years) | Hospitals (10) | Children of HIV infected mothers (0 to 14 years) | Infected children with echocardiographic evaluation available (5 years follow-up) | Congestive HF | 199 (after excluding prevalent cases) | NR | NR | 14 | | 14 (cumulative incidence)  7.0 (incidence) |
|  |  |  |  | Infants of HIV infected mothers (0 to <28 days) | Infected Infants (5 years follow-up) | Congestive HF | 93 | NR | NR | 4 | | 5.1(cumulative incidence)  4.3 (incidence) |
|  |  |  |  |  | Uninfected Infants(5 years follow-up) | Congestive HF | 463 | NR | NR | 1 | | 0.2(cumulative -incidence  0.2 (incidence) |
| **Pneumonia** | | | | | | | | | | | | |
| llten 2003[73] | Prospective | Turkey, NR | Hospital (1) | Acute pneumonia, 2 to 24 months |  | Congestive HF | 50 | 14 | 28 | 7 | | 14 |
| Abbreviations: HF: Heart Failure; HIV: Human Immunodeficiency Virus; NR: Not Reported.*= calculated data from the study, ^#^ After removing the prevalent cases. | | | | | | | | | | | | |

**Table C2: Prevalence of HF in all non CVD studies**

| **Study name** | **Study design** | **Country, Period** | **Setting** | **Study population, (age range)** | **Subgroups** | **Type of HF** | **Sample size** | **Gender** | | **HF prevalence and distribution in study sub-groups** | | |
| --- | --- | --- | --- | --- | --- | --- | --- | --- | --- | --- | --- | --- |
|  |  |  |  |  |  |  |  | **Female (n)** | **Female (%)** | **Cases (n)** | **Prevalence (%)** | **Distribution of prevalent cases of HF in study subgroups (%)** |
| **Renal Disorders** | | | | | | | | | | | | |
| Gunasekaran 2015 ^[74]^ | Prospective | India, 2013 to 2014 (1 year and 6 months) | Hospital (1) | ANS, (1 to 13 years) | PIGN | Congestive HF | 72 | 32* | 44.4* | 8 | 11.1 | - |
|  |  |  |  | PIGN | PSGN | Congestive HF | 65 | 30* | 46.1* | 8 | 12.3 | - |
| Wong 2013 [75] | Prospective | New Zealand, 2007 to 2009 (2 years) | Hospitals (country wide) | APSGN (definite/ probable),(1.4 to 14.7 years) |  | Congestive HF | 176 | 62 | 35.2* | 8 | 4.5* | - |
| Krishnamurthy 2013[76] | Prospective | India, 2010 to 2011 (10 months) | Hospital (1) | AKI, (1 to 144 months) |  | Congestive HF | 54 | 25 | 46.3 | 2 | 3.8 | - |
| Vachvanichsanong 2012[77] | Retrospective | Thailand, 1984 to 2007 (26 years) | Hospital (1) | AKI ,(0 to 30 days) | All patients | Congestive HF | 139 | 51 | 36.7* | 17* | 12.2* | - |
|  |  |  |  |  | Aged 0 to 2 days | Congestive HF | 54 | NR | NR | 4 | 7.4* | - |
|  |  |  |  |  | Aged 3 to 7 days | Congestive HF | 37 | NR | NR | 2 | 5.4* | - |
|  |  |  |  |  | Aged 8 to 14 days | Congestive HF | 24 | NR | NR | 7 | 29.2* | - |
|  |  |  |  |  | Aged 15 to 30 days | Congestive HF | 24 | NR | NR | 4 | 16.7* | - |
| Vachvanichsanong 2006 [78] | Retrospective | Thailand, 1982 to 2004 (22 years and 10 months) | Hospital (1) | Acute renal failure, (1 month to 16.7 years) |  | HF | 311 | NR | NR | 26 | 8.4 | - |
| Becquet 2010[79] | Retrospective | France(French Polynesia), 2005 to 2007 (3 years) | Hospital (1) | Acute PSGN,(0 to <15 years) |  | HF | 50 | 23 | 46 | 7 | 14.0 | - |
| Duzova 2010[80] | Prospective | Turkey , 2006 to 2007 (1 year) | Hospitals (17) | AKI ,(1 to 18 years) | New born (<1 month) | HF | 154 | NR | NR | 15 | 9.7 | - |
| Sarkissian 1997 [81] | Prospective | Armenia, 1992 to 1996 (5 years) | Hospital (1) | APGN, (1 to <16 years) |  | Congestive HF | 474 | 166* | 35* | 45 | 10.0 | - |
| Olowu 2002[82] | Retrospective | Nigeria, 1994 to 1997 (3 years) | Hospital (1) | Acute glomerulonephritis, (0.6 to 12 years) |  | HF | 29 | 13 | 44.8* | 7 | 24.1* | - |
| **HIV/AIDS** | | | | | | | | | | | | |
| Dimitriu 2014[83], Dimitriu 2009[84] | Prospective | Romania, NR | Hospital (1) | HIV/ AIDS, (2 to 16 years) |  | HF | 51 | NR | NR | 11 | 21.6 | - |
| Okoromah 2012[85] | Case-control | Nigeria, 2004 to 2007 (3 years) | Hospital (1) | HIV positive, (18 to 144 months) |  | Congestive HF | 83 | NR | NR | 10 | 12.0 | - |
|  |  |  | Community and hospital | HIV negative, (18 to 144 months) |  | Congestive HF | 83 | NR | NR | 0 | 0 | - |
| Herdy 2003[86] | Prospective | Brazil, 1993 to 2000 (8 years) | Hospital (1) | HIV, (4 months to 12 years) | All patients | Congestive HF | 47 | NR | NR | 8 | 17.0 | - |
|  |  |  |  |  | Group 1- Zidovudine treated | Congestive HF | 20 | NR | NR | 5 | 25.0 | - |
|  |  |  |  |  | Group 2- Zidovudine alone (initially) later with a combination of other drugs (diagnosed before December 1995) | Congestive HF | 10 | NR | NR | 2 | 20.0 | - |
|  |  |  |  |  | Group 3- Combination of 2 or 3 antiretroviral drugs since the beginning (diagnosed after January 1996) | Congestive HF | 17 | NR | NR | 1 | 6.0 | - |
| Cunha 2008[87] | Retrospective | Brazil, 1990 to 2002 (13 years) | Hospital (1) | AIDS, (0 to <13years) |  | Congestive HF | 93 | 47 | 50.5 | 12 | 12.9* | - |
| Diogenes 2005[88] | Prospective | Brazil, 1996 to 2004 (8 years) | Hospital (NR) | HIV-1 ,(13 days to 13 years) | HIV infected | Congestive HF | 41 | NR | NR | 12 | 29.3* | - |
|  |  |  |  |  | HIV seroconverted | Congestive HF | 43 | NR | NR | 0 | 0 | - |
|  |  |  |  |  | Dilated cardiomyopathy (as etiology for congestive HF) | Congestive HF | 12 | NR | NR | 5 |  | 41.7* |
|  |  |  |  |  | Pericardial effusion with cardiac tamponade caused by bacterial pericarditis (as etiology for congestive HF) | Congestive HF | 12 | NR | NR | 2 |  | 16.7* |
|  |  |  |  |  | Pulmonary hypertension secondary to chronic lung disease (as etiology for congestive HF) | Congestive HF | 12 | NR | NR | 3 |  | 25.0* |
|  |  |  |  |  | Respiratory infection, sepsis, and anemia (as etiology for congestive HF) | Congestive HF | 12 | NR | NR | 3 |  | 25.0* |
| Starc 2002[69], Fisher 2005[70]  Lipshultz 1998 [71]  Starc 1999[72] | Prospective | US, 1990 to Jan 1997 (6 years) | Hospitals (10) | Children of HIV infected mothers (0 to 14 years) |  | Congestive HF | 201 | NR | NR | 2 | 1.0 | - |
| **Hematology/ Oncology** | | | | | | | | | | | | |
| Karimi 2011[89] | Cross-sectional | Iran, 2007 to 2010 (3 years) | Hospital (1) | BTM, (1 to 15 years) | All patients | Congestive HF | 328 | NR | NR | 47* | 14.3* | - |
|  |  |  |  |  | Aged 1 to 5 years | Congestive HF | 46 | NR | NR | 1 | 2.2* | - |
|  |  |  |  |  | Aged 6 to 10 years | Congestive HF | 89 | NR | NR | 3 | 3.4* | - |
|  |  |  |  |  | Aged 11 to 15 years | Congestive HF | 193 | NR | NR | 43 | 22.3* | - |
| **Other conditions** | | | | | | | | | | | | |
| Lagunju 2005[90] | Retrospective | Nigeria, 2000 to 2004 (5 years) | Hospital (1) | Measles, (4 months to 10 years) |  |  | 666 | 319 | 47.9 | 2 | 0.3 | - |
| Ahmed 2011[91] | Retrospective | Scotland, 2002 to 2008 (6 years) | Hospital (1) | Vitamin D deficiency, (2 weeks to 14 years) |  | HF | 160 | 77 | 48.1 | 1 | 0.6 | - |
| Li 2009[92] | Retrospective | China, 1990 to 2007 (17 years and 6 months) | Hospital (1) | Foreign body aspiration, (8 months to 8 years) |  | HF | 38 | 17 | 44.7 | 2 | 5.3 | - |
| Camilla 2008[93] | Cross-sectional (Pt prevalence) | Italy | Community | Organ failure, (0 to <18years) | All inhabitants | CHF | 647,727 | NR | NR | 21 | 0.0032* |  |
|  |  |  |  |  | DCM (as etiology) |  | 21 | NR | NR | 13 | - | 62* |
|  |  |  |  |  | Surgically trait congenital cardiopathy |  | 21 | NR | NR | 7 | - | 33* |
|  |  |  |  |  | Not surgically  trait congenital cardiopathy |  | 21 | NR | NR | 1 | - | 15* |
|  |  |  |  |  | Severe (instrumental evaluation) |  | 21 | NR | NR | 5 | - | 24 |
|  |  |  |  |  | Moderate (instrumental evaluation) |  | 21 | NR | NR | 7 | - | 33 |
|  |  |  |  |  | Mild (instrumental evaluation) |  | 21 | NR | NR | 9 | - | 43 |
|  |  |  |  |  | Severe (clinical criteria) |  | 21 | NR | NR | 2 | - | 10 |
|  |  |  |  |  | Moderate (clinical criteria) |  | 21 | NR | NR | 4 | - | 19 |
|  |  |  |  |  | Mild (clinical criteria) |  | 21 | NR | NR | 7 | - | 33 |
|  |  |  |  |  | Asymptomatic (clinical criteria |  | 21 | NR | NR | 8 | - | 38 |
| Abbreviations: ANS; Acute nephrotic syndrome; AIDS, acquired immunodeficiency syndrome; AKI, acute kidney injury; APGN, acute post-infectious glomerulonephritis; APSGN, acute post-streptococcal glomerulonephritis; BTM, β-thalassemia major; CHF, chronic heart failure; CVD, cardiovascular disease; DCM, dilated cardiomyopathy; HF, heart failure; HIV, human immunodeficiency virus; NR, not reported; PIGN, post-infectious glomerulonephritis; PSGN, post-streptococcal glomerulonephritis.*Calculated data from source article | | | | | | | | | | | | |

# References

1 Massin MM, Astadicko I, Dessy H (2008) Epidemiology of heart failure in a tertiary pediatric center. Clin Cardiol 31: 388-391

2 Andrews RE, Fenton MJ, Ridout DA, Burch M (2008) New-onset heart failure due to heart muscle disease in childhood: a prospective study in the United kingdom and Ireland. Circulation 117: 79-84

3 Neumann T, Biermann J, Erbel R, Neumann A, Wasem J, Ertl G, Dietz R (2009) Heart failure: the commonest reason for hospital admission in Germany: medical and economic perspectives. Dtsch Arztebl Int 106: 269-275

4 Schmidt S, Hendricks V, Griebenow R, Riedel R (2013) Demographic change and its impact on the health-care budget for heart failure inpatients in Germany during 1995-2025. Herz 38: 862-867

5 Tseng CH (2010) The age- and sex-specific incidence and medical expenses of heart failure hospitalization in 2005 in Taiwan: a study using data from the National Health Insurance. J Am Geriatr Soc 58: 611-613

6 Jimenez-Garcia R, Esteban-Vasallo MD, Rodriguez-Rieiro C, Hernandez-Barrera V, Dominguez-Berjon MA, Carrasco Garrido P, Lopez de Andres A, Cameno Heras M, Iniesta Fornies D, Astray-Mochales J (2014) Coverage and predictors of vaccination against 2012/13 seasonal influenza in Madrid, Spain: analysis of population-based computerized immunization registries and clinical records. Hum Vaccin Immunother 10: 449-455

7 Rodriguez-Rieiro C, Dominguez-Berjon MF, Esteban-Vasallo MD, Sanchez-Perruca L, Astray-Mochales J, Fornies DI, Ordonez DB, Jimenez-Garcia R (2010) Vaccination coverage against 2009 seasonal influenza in chronically ill children and adults: analysis of population registries in primary care in Madrid (Spain). Vaccine 28: 6203-6209

8 Lagunju IA, Omokhodion SI (2003) Childhood heart failure in Ibadan. West Afr J Med 22: 42-45

9 Adekanmbi AF, Ogunlesi TA, Olowu AO, Fetuga MB (2007) Current trends in the prevalence and aetiology of childhood congestive cardiac failure in Sagamu. J Trop Pediatr 53: 103-106

10 Oyedeji OA, Oluwayemi IO, Oyedeji AT (2010) Heart Failure in Nigerian Children. The Cardiology 5: 18-22

11 Animasahun A, Itiola J, Falase B (2015) Congestive Cardiac Failure among Nigerian Children; Pattern and Outcome. Int Cardiovasc Res J 9: 164-168

12 Najm HK, Williams WG, Coles JG, Rebeyka IM, Freedom RM (1996) Scimitar syndrome: twenty years' experience and results of repair. J Thorac Cardiovasc Surg 112: 1161-1168; discussion 1168-1169

13 Tomlinson TW, Scott CH, Trotman HL (2010) Congenital cardiovascular lesions in children with trisomy 21 at the Bustamante Hospital for Children. Cardiol Young 20: 327-331

14 Hong SJ, Choi HJ, Kim YH, Hyun MC, Lee SB, Cho JY (2012) Clinical features and surgical outcomes of complete transposition of the great arteries. Korean J Pediatr 55: 377-382

15 Rialon KL, Murillo R, Fevurly RD, Kulungowski AM, Zurakowski D, Liang M, Kozakewich HP, Alomari AI, Fishman SJ (2015) Impact of Screening for Hepatic Hemangiomas in Patients with Multiple Cutaneous Infantile Hemangiomas. Pediatr Dermatol 32: 808-812

16 Murtuza B, Fenton M, Burch M, Gupta A, Muthialu N, Elliott MJ, Hsia TY, Tsang VT, Kostolny M (2013) Pediatric heart transplantation for congenital and restrictive cardiomyopathy. Ann Thorac Surg 95: 1675-1684

17 LaPage M, Rhee EK, Canter CE (2010) Tachyarrhythmias after pediatric heart transplantation. J Heart Lung Transplant 29: 273 -277

18 Marom D, Ashkenazi S, Samra Z, Birk E (2013) Infective endocarditis in previously healthy children with structurally normal hearts. Pediatr Cardiol 34: 1415-1421

19 Meberg A, Otterstad JE, Froland G, Hals J, Sorland SJ (1999) Early clinical screening of neonates for congenital heart defects: the cases we miss. Cardiol Young 9: 169-174

20 Vaidyanathan B, Nair SB, Sundaram KR, Babu UK, Shivaprakasha K, Rao SG, Kumar RK (2008) Malnutrition in children with congenital heart disease (CHD) determinants and short term impact of corrective intervention. Indian Pediatr 45: 541-546

21 Okoromah CA, Ekure EN, Lesi FE, Okunowo WO, Tijani BO, Okeiyi JC (2011) Prevalence, profile and predictors of malnutrition in children with congenital heart defects: a case-control observational study. Arch Dis Child 96: 354-360

22 Sadoh WE, Osarogiagbon WO (2013) Underlying congenital heart disease in Nigerian children with pneumonia. Afr Health Sci 13: 607-612

23 Shah GS, Singh MK, Pandey TR, Kalakheti BK, Bhandari GP (2008) Incidence of congenital heart disease in tertiary care hospital. Kathmandu Univ Med J (KUMJ) 6: 33-36

24 Miyake T, Shinohara T, Nakamura Y, Fukuda T, Tasato H, Toyohara K, Tanihira Y (2004) Spontaneous closure of ventricular septal defects followed up from <3 months of age. Pediatr Int 46: 135-140

25 Sadoh WE (2010) Natural history of ventricular septal defects in Nigerian children. South African Journal of Child Health 4: 16 -19

26 Najm HK, Williams WG, Chuaratanaphong S, Watzka SB, Coles JG, Freedom RM (1998) Primum atrial septal defect in children: early results, risk factors, and freedom from reoperation. Ann Thorac Surg 66: 829-835

27 Azhari N, Shihata MS, Al-Fatani A (2004) Spontaneous closure of atrial septal defects within the oval fossa. Cardiol Young 14: 148-155

28 Trivedi KR, Karamlou T, Yoo SJ, Williams WG, Freedom RM, McCrindle BW (2006) Outcomes in 45 children with ductal origin of the distal pulmonary artery. Ann Thorac Surg 81: 950-957

29 Harshangi SV, Itagi LN, Patil V (2013) Clinical study of congenital heart disease in infants in tertiary care hospital Journal of Pharmacy and Scientific Innovation 2:

30 Agny M, Cobanoglu A (1999) Repair of partial atrioventricular septal defect in children less than five years of age: late results. Ann Thorac Surg 67: 1412-1414

31 Yim D, Yong MS, d'Udekem Y, Brizard CP, Konstantinov IE (2015) Early Surgical Repair of the Coronary Artery Fistulae in Children: 30 Years of Experience. Ann Thorac Surg 100: 188-194

32 Mottin B, Baruteau A, Boudjemline Y, Piéchaud J-F, Godart F, Guérin P (2013) Percutaneous closure of coronary artery fistulas in pediatrics. Archives of Cardiovascular Disease 106: 465-467

33 Parvathy U, Balakrishnan KR, Ranjith MS, Saldanha R, Sai S, Vakamudi M (2000) Surgical experience with congenital heart disease in Down's syndrome. Indian Heart J 52: 438-441

34 Adeeb SM, Leman H, Sallehuddin A, Yakub A, Awang Y, Alwi M (2004) Coarctation of aorta repair at the National Heart Institute (1983-1994). Med J Malaysia 59: 11-14

35 Skinner JR, Manzoor A, Hayes AM, Joffe HS, Martin RP (1997) A regional study of presentation and outcome of hypertrophic cardiomyopathy in infants. Heart 77: 229-233

36 Nugent AW, Daubeney PE, Chondros P, Carlin JB, Cheung M, Wilkinson LC, Davis AM, Kahler SG, Chow CW, Wilkinson JL, Weintraub RG (2003) The epidemiology of childhood cardiomyopathy in Australia. N Engl J Med 348: 1639-1646

37 Wilkinson JD, Westphal JA, Bansal N, Czachor JD, Razoky H, Lipshultz SE (2015) Lessons learned from the Pediatric Cardiomyopathy Registry (PCMR) Study Group. Cardiol Young 25 Suppl 2: 140-153

38 Alvarez JA, Orav EJ, Wilkinson JD, Fleming LE, Lee DJ, Sleeper LA, Rusconi PG, Colan SD, Hsu DT, Canter CE, Webber SA, Cox GF, Jefferies JL, Towbin JA, Lipshultz SE (2011) Competing risks for death and cardiac transplantation in children with dilated cardiomyopathy: results from the pediatric cardiomyopathy registry. Circulation 124: 814-823

39 Webber SA, Lipshultz SE, Sleeper LA, Lu M, Wilkinson JD, Addonizio LJ, Canter CE, Colan SD, Everitt MD, Jefferies JL, Kantor PF, Lamour JM, Margossian R, Pahl E, Rusconi PG, Towbin JA (2012) Outcomes of restrictive cardiomyopathy in childhood and the influence of phenotype: a report from the Pediatric Cardiomyopathy Registry. Circulation 126: 1237-1244

40 Colan SD, Lipshultz SE, Lowe AM, Sleeper LA, Messere J, Cox GF, Lurie PR, Orav EJ, Towbin JA (2007) Epidemiology and cause-specific outcome of hypertrophic cardiomyopathy in children: findings from the Pediatric Cardiomyopathy Registry. Circulation 115: 773-781

41 Towbin JA, Lowe AM, Colan SD, Sleeper LA, Orav EJ, Clunie S, Messere J, Cox GF, Lurie PR, Hsu D, Canter C, Wilkinson JD, Lipshultz SE (2006) Incidence, causes, and outcomes of dilated cardiomyopathy in children. JAMA 296: 1867-1876

42 Everitt MD, Sleeper LA, Lu M, Canter CE, Pahl E, Wilkinson JD, Addonizio LJ, Towbin JA, Rossano J, Singh RK, Lamour J, Webber SA, Colan SD, Margossian R, Kantor PF, Jefferies JL, Lipshultz SE (2014) Recovery of echocardiographic function in children with idiopathic dilated cardiomyopathy: results from the pediatric cardiomyopathy registry. J Am Coll Cardiol 63: 1405-1413

43 Marcos-Alonso S, Vázquez-García L, Fernández Santamarina I, López Abel B, Martínez Soto S, Rey García S, Fontenla García J, Hurtado Díaz J, Herrero Hermida J, Luaces González J, Fuentes Carballal J, F. RN Cardiomyopathy in a pediatric population: Key results from 10 years. P-24 presented at the 47th Annual Meeting of the Association for European Pediatric and Congenital Cardiology, May 22-25, 2013. .

44 Tsirka AE, Trinkaus K, Chen SC, Lipshultz SE, Towbin JA, Colan SD, Exil V, Strauss AW, Canter CE (2004) Improved outcomes of pediatric dilated cardiomyopathy with utilization of heart transplantation. J Am Coll Cardiol 44: 391-397

45 Soongswang J, Sangtawesin C, Sittiwangkul R, Wanitkun S, Muangmingsuk S, Sopontammarak S, Klungratana C, Kangkagate C (2002) Myocardial diseases in Thai children. J Med Assoc Thai 85 Suppl 2: S648-657

46 Saji T, Matsuura H, Hasegawa K, Nishikawa T, Yamamoto E, Ohki H, Yasukochi S, Arakaki Y, Joo K, Nakazawa M (2012) Comparison of the clinical presentation, treatment, and outcome of fulminant and acute myocarditis in children. Circ J 76: 1222-1228

47 Bejiqi R, Retkoceri R, Zeka N (2013) Children with congenital heart disease and sideropenic anaemia less than one year in comparison with healthy children seen in kosova. International Journal of Cardiology Conference: 9th International Congress of Update in Cardiology and Cardiovascular Surgery Antalya Turkey Conference Publication: (varpagings) 163 (3 SUPPL. 1) pp S67

48 Orun UA, Ceylan O, Bilici M, Karademir S, Ocal B, Senocak F, Ozgur S, Dogan V, Yilmaz O, Keskin M (2012) Acute rheumatic fever in the Central Anatolia Region of Turkey: a 30-year experience in a single center. Eur J Pediatr 171: 361-368

49 Karaaslan S, Oran B, Reisli I, Erkul I (2000) Acute rheumatic fever in Konya, Turkey. Pediatr Int 42: 71-75

50 da Silva CH (1999) Rheumatic fever: a multicenter study in the state of Sao Paulo. Pediatric Committee--Sao Paulo Pediatric Rheumatology Society. Rev Hosp Clin Fac Med Sao Paulo 54: 85-90

51 Qurashi MA (2009) The pattern of acute rheumatic fever in children: Experience at the children's hospital, Riyadh, Saudi Arabia. J Saudi Heart Assoc 21: 215-220

52 Rayamajhi A, Sharma D, Shakya U (2007) Clinical, laboratory and echocardiographic profile of acute rheumatic fever in Nepali children. Ann Trop Paediatr 27: 169-177

53 Prakoso R, Roebiono PS, Lilyasar iO (2014) Incidence and pattern of rheumatic heart disease among children at National Cardiovascular Center Harapan Kita, Jakarta. Annals of Pediatric Cardiology Conference: 5th Congress of the Asia-Pacific Pediatric Cardiac Society, APPCS 2014 New Delhi India Conference Publication: (varpagings) 7: pp S42-S43

54 Thakur JS, Negi PC, Ahluwalia SK, Vaidya NK (1996) Epidemiological survey of rheumatic heart disease among school children in the Shimla Hills of northern India: prevalence and risk factors. J Epidemiol Community Health 50: 62-67

55 Gapu P, Bwakura-Dangarembizi M, Kandawasvika G, Kao D, Bannerman C, Hakim J, Matenga JA (2015) Rheumatic fever and rheumatic heart disease among children presenting to two referral hospitals in Harare, Zimbabwe. S Afr Med J 105: 384-388

56 Bitar FF, Hayek P, Obeid M, Gharzeddine W, Mikati M, Dbaibo GS (2000) Rheumatic fever in children: a 15-year experience in a developing country. Pediatr Cardiol 21: 119-122

57 Lertsapcharoen P, Khongphatthanayothin A, Chotivittayatarakorn P, Thisyakorn C, Pathmanand C, Sueblinvong V (2005) Infective endocarditis in pediatric patients: an eighteen-year experience from King Chulalongkorn Memorial Hospital. J Med Assoc Thai 88 Suppl 4: S12-16

58 Sadiq M, Nazir M, Sheikh SA (2001) Infective endocarditis in children--incidence, pattern, diagnosis and management in a developing country. Int J Cardiol 78: 175-182

59 Caldarelli M, Di Rocco C, Iannelli A, Rollo M, Tamburrini G, Velardi F (1997) Combined management of intracranial vascular malformations in children. J Neurosurg Sci 41: 315-324

60 Massin MM, Benatar A, Rondia G (2008) Epidemiology and outcome of tachyarrhythmias in tertiary pediatric cardiac centers. Cardiology 111: 191-196

61 Estepa R, Gallego N, Orte L, Puras E, Aracil E, Ortuno J (2001) Renovascular hypertension in children. Scand J Urol Nephrol 35: 388-392

62 Jozwiak S, Kotulska K, Kasprzyk-Obara J, Domanska-Pakiela D, Tomyn-Drabik M, Roberts P, Kwiatkowski D (2006) Clinical and genotype studies of cardiac tumors in 154 patients with tuberous sclerosis complex. Pediatrics 118: e1146-1151

63 Borzouee M, Jannati M (2008) Distribution and Characteristics of the Heart Disease in Pediatric Age Group in Southern Iran. International Cardivascular Research Journal 2: 48-51

64 Kayalı S, Dogan V, Yoldas T, Kaya Ö, Özgür S, Ertugrul I, Koç M, Arman Örün U, Karademir S (2015) Primary cardiac tumors: single center experience. Am J Cardiol 115: S163

65 Benzecry SG, Leite HP, Oliveira FC, Santana EMJF, de Carvalho WB, Silva CM (2008) Interdisciplinary approach improves nutritional status of children with heart diseases. Nutrition 24: 669-674

66 van Dalen EC, van der Pal HJ, Kok WE, Caron HN, Kremer LC (2006) Clinical heart failure in a cohort of children treated with anthracyclines: a long-term follow-up study. Eur J Cancer 42: 3191-3198

67 Berrak SG, Ewer MS, Jaffe N, Pearson P, Ried H, Zietz HA, Benjamin RS (2001) Doxorubicin cardiotoxicity in children: reduced incidence of cardiac dysfunction associated with continuous-infusion schedules. Oncol Rep 8: 611-614

68 Godoy LY, Fukushige J, Igarashi H, Matsuzaki A, Ueda K (1997) Anthracycline-induced cardiotoxicity in children with malignancies. Acta Paediatr Jpn 39: 188-193

69 Starc TJ, Lipshultz SE, Easley KA, Kaplan S, Bricker JT, Colan SD, Lai WW, Gersony WM, Sopko G, Moodie DS, Schluchter MD (2002) Incidence of cardiac abnormalities in children with human immunodeficiency virus infection: The prospective P2C2 HIV study. J Pediatr 141: 327-334

70 Fisher SD, Easley KA, Orav EJ, Colan SD, Kaplan S, Starc TJ, Bricker JT, Lai WW, Moodie DS, Sopko G, Lipshultz SE (2005) Mild dilated cardiomyopathy and increased left ventricular mass predict mortality: the prospective P2C2 HIV Multicenter Study. Am Heart J 150: 439-447

71 Lipshultz SE, Easley KA, Orav EJ, Kaplan S, Starc TJ, Bricker JT, Lai WW, Moodie DS, McIntosh K, Schluchter MD, Colan SD (1998) Left ventricular structure and function in children infected with human immunodeficiency virus: the prospective P2C2 HIV Multicenter Study. Pediatric Pulmonary and Cardiac Complications of Vertically Transmitted HIV Infection (P2C2 HIV) Study Group. Circulation 97: 1246-1256

72 Starc TJ, Lipshultz SE, Kaplan S, Easley KA, Bricker JT, Colan SD, Lai WW, Gersony WM, Sopko G, Moodie DS, Schluchter MD (1999) Cardiac complications in children with human immunodeficiency virus infection. Pediatric Pulmonary and Cardiac Complications of Vertically Transmitted HIV Infection (P2C2 HIV) Study Group, National Heart, Lung, and Blood Institute. Pediatrics 104: e14

73 Ilten F, Senocak F, Zorlu P, Tezic T (2003) Cardiovascular changes in children with pneumonia. Turk J Pediatr 45: 306-310

74 Gunasekaran K, Krishnamurthy S, Mahadevan S, Harish BN, Kumar AP (2015) Clinical Characteristics and Outcome of Post-Infectious Glomerulonephritis in Children in Southern India: A Prospective Study. Indian J Pediatr 82: 896-903

75 Wong W, Lennon DR, Crone S, Neutze JM, Reed PW (2013) Prospective population-based study on the burden of disease from post-streptococcal glomerulonephritis of hospitalised children in New Zealand: epidemiology, clinical features and complications. J Paediatr Child Health 49: 850-855

76 Krishnamurthy S, Narayanan P, Prabha S, Mondal N, Mahadevan S, Biswal N, Srinivasan S (2013) Clinical profile of acute kidney injury in a pediatric intensive care unit from Southern India: A prospective observational study. Indian J Crit Care Med 17: 207-213

77 Vachvanichsanong P, McNeil E, Dissaneevate S, Dissaneewate P, Chanvitan P, Janjindamai W (2012) Neonatal acute kidney injury in a tertiary center in a developing country. Nephrol Dial Transplant 27: 973-977

78 Vachvanichsanong P, Dissaneewate P, Lim A, McNeil E (2006) Childhood acute renal failure: 22-year experience in a university hospital in southern Thailand. Pediatrics 118: e786-791

79 Becquet O, Pasche J, Gatti H, Chenel C, Abely M, Morville P, Pietrement C (2010) Acute post-streptococcal glomerulonephritis in children of French Polynesia: a 3-year retrospective study. Pediatr Nephrol 25: 275-280

80 Duzova A, Bakkaloglu A, Kalyoncu M, Poyrazoglu H, Delibas A, Ozkaya O, Peru H, Alpay H, Soylemezoglu O, Gur-Guven A, Bak M, Bircan Z, Cengiz N, Akil I, Ozcakar B, Uncu N, Karabay-Bayazit A, Sonmez F (2010) Etiology and outcome of acute kidney injury in children. Pediatr Nephrol 25: 1453-1461

81 Sarkissian A, Papazian M, Azatian G, Arikiants N, Babloyan A, Leumann E (1997) An epidemic of acute postinfectious glomerulonephritis in Armenia. Arch Dis Child 77: 342-344

82 Olowu WA (2002) Systemic complications of acute glomerulonephritis in Nigerian children. Niger Postgrad Med J 9: 83-87

83 Dimitriu AG, Jitareanu C, Dimitriu L (2014) Cardiac involvement, major problem in human immunodeficiency virus infection (HIV) in children. Archives of Disease in Childhood Conference: 5th Congress of the European Academy of Paediatric Societies, EAPS 2014 Barcelona Spain Conference Start: 20141017 Conference End: 20141021 Conference Publication: (varpagings) Date of Publication: 99 (pp A320), 2014.

84 Dimitriu AG and Jitareanu C. .European Heart Journal. Conference: (2009) Peculiar aspects of cardiac involvement in children with acquired immunodeficiency syndrome. European Society of Cardiology, ESC Congress 2009 Barcelona Spain Conference Start: 20090829 Conference End: 20090902 Conference Publication: (varpagings) 2009: 30 (pp 1004)

85 Okoromah CAN, Ojo OO, Ogunkunle OO (2012) Cardiovascular dysfunction in human immunodeficiency virus (HIV)-infected children in a sub-saharan african country: Comparative cross-sectional observational Study. Journal of Tropical Pediatrics 58: 3-11

86 Herdy GV, Pinto CA, Lopes VG, Ribeiro RP, Gomes IM, Tchou HY, Melo R, Kurdian B, Junior Tavares Pde A (2003) Study of the cardiac alterations in HIV-infected children consequent to the antiretroviral therapy. Prospective study of 47 cases. Arq Bras Cardiol 80: 311-320

87 Cunha Mdo C, Siqueira Filho AG, Santos SR, Abreu TF, Oliveira RH, Baptista DM, Dantas MC, Carvalho MF, Guedes LG (2008) AIDS in childhood: cardiac involvement with and without triple combination antiretroviral therapy. Arq Bras Cardiol 90: 11-17

88 Diogenes MS, Succi RC, Machado DM, Moises VA, Novo NF, Carvalho AC (2005) [Cardiac longitudinal study of children perinatally exposed to human immunodeficiency virus type 1]. Arq Bras Cardiol 85: 233-240

89 Karimi M, Emadmarvasti V, Hoseini J, Shoja L (2011) Major causes of hospital admission in Beta thalassemia major patients in southern iran. Iran J Pediatr 21: 509-513

90 Lagunju IA, Orimadegun AE, Oyedemi DG (2005) Measles in Ibadan: a continuous scourge. Afr J Med Med Sci 34: 383-387

91 Ahmed SF, Franey C, McDevitt H, Somerville L, Butler S, Galloway P, Reynolds L, Shaikh MG, Wallace AM (2011) Recent trends and clinical features of childhood vitamin D deficiency presenting to a children's hospital in Glasgow. Arch Dis Child 96: 694-696

92 Li Y, Wu W, Yang X, Li J (2009) Treatment of 38 cases of foreign body aspiration in children causing life-threatening complications. Int J Pediatr Otorhinolaryngol 73: 1624-1629

93 Camilla R, Magnetti F, Barbera C, Bignamini E, Riggi C, Coppo R (2008) Children with chronic organ failure possibly ending in organ transplantation: a survey in an Italian region of 5,000,000 inhabitants. Acta Paediatr 97: 1285-1291
